# Bacterial Gamma-Glutamyl Transpeptidase, an Emerging Biocatalyst: Insights Into Structure–Function Relationship and Its Biotechnological Applications

**DOI:** 10.3389/fmicb.2021.641251

**Published:** 2021-04-09

**Authors:** Meenu Saini, Amuliya Kashyap, Shruti Bindal, Kuldeep Saini, Rani Gupta

**Affiliations:** Department of Microbiology, University of Delhi South Campus, New Delhi, India

**Keywords:** autoprocessing, bacteria, catalytic mechanism, γ-glutamyl transpeptidase, γ-glutamyl peptides, glutathione, poly-γ-glutamic acid

## Abstract

Gamma-glutamyl transpeptidase (GGT) enzyme is ubiquitously present in all life forms and plays a variety of roles in diverse organisms. Higher eukaryotes mainly utilize GGT for glutathione degradation, and mammalian GGTs have implications in many physiological disorders also. GGTs from unicellular prokaryotes serve different physiological functions in Gram-positive and Gram-negative bacteria. In the present review, the physiological significance of bacterial GGTs has been discussed categorizing GGTs from Gram-negative bacteria like *Escherichia coli* as glutathione degraders and from pathogenic species like *Helicobacter pylori* as virulence factors. Gram-positive bacilli, however, are considered separately as poly-γ-glutamic acid (PGA) degraders. The structure–function relationship of the GGT is also discussed mainly focusing on the crystallization of bacterial GGTs along with functional characterization of conserved regions by site-directed mutagenesis that unravels molecular aspects of autoprocessing and catalysis. Only a few crystal structures have been deciphered so far. Further, different reports on heterologous expression of bacterial GGTs in *E. coli* and *Bacillus subtilis* as hosts have been presented in a table pointing toward the lack of fermentation studies for large-scale production. Physicochemical properties of bacterial GGTs have also been described, followed by a detailed discussion on various applications of bacterial GGTs in different biotechnological sectors. This review emphasizes the potential of bacterial GGTs as an industrial biocatalyst relevant to the current switch toward green chemistry.

## Introduction

The enzyme γ-glutamyl transpeptidase (GGT; E.C.2.3.3.2) is conserved throughout all three domains of life, ranging from single-celled prokaryotes to multicellular higher eukaryotes (Rawlings et al., 2010). It belongs to the N-terminal nucleophile hydrolase superfamily and exists as a heterodimer of a large and a small subunit (Nash and Tate, 1984; Suzuki et al., 1989; Brannigan et al., 1995). It is a two-substrate enzyme catalyzing the transfer of γ-glutamyl moiety from donor substrates, such as glutathione/glutamine, to an acceptor substrate, which can be water (hydrolysis) or other amino acids/small peptides (transpeptidation) or the donor itself (autotranspeptidation) (Thompson and Meister, 1977; Tate and Meister, 1981). Here, the GGT is commonly referred to by its old name as “gamma-glutamyl transpeptidase;” however, according to the Nomenclature Committee of International Union of Biochemistry and Molecular biology, its accepted name is γ-glutamyltransferase (Balakrishna and Prabhune, 2014).

Gamma-glutamyl transpeptidase enzyme was first reported in mammals, such as sheep and rat (Hanes et al., 1952; Tate and Meister, 1976). It is worldwide recognized as a diagnostic marker for many physiological diseases in humans (Yamada et al., 1991; Whitfield, 2001; Grundy, 2007). Although its existence in the living world was known for a long time, only after its discovery in prokaryotes led to a profound understanding of its structure–function relationship and also suggested its application in the synthesis of various γ-glutamyl compounds. Presently, it finds biotechnological significance in the synthesis of various bioactive γ-glutamyl compounds for application in food and pharmaceutical sectors (Suzuki et al., 2004a; Wang et al., 2008; Shuai et al., 2010; Saini et al., 2017; Mu et al., 2019).

Mammalians GGTs have already been extensively reviewed (Tate and Meister, 1981; Taniguchi and Ikeda, 1998; Chikhi et al., 1999; Whitfield, 2001). Microbial GGTs, covering the structure–function relationship of the protein and their biotechnological significance, have been reviewed by Castellano and Merlino (2012) and subsequently by Balakrishna and Prabhune (2014).

The current review focuses mainly on bacterial GGTs with emphasis on their physiological significance, structural and molecular aspects, autoprocessing, and enzyme catalysis along with their relevance in biotechnology and biomedicine sectors.

## Diversity of Bacterial GGTs

Gamma-glutamyl transpeptidases diversity ranging from microbes to mammals has been described by Verma et al. (2015) through a phylogenetic tree, including 47 diverse GGT sequences. This phylogenetic tree had two main branches and various clades (Verma et al., 2015). The tree also showed some interesting evolutionary relationships of prokaryotic and eukaryotic GGTs, as it grouped pathogenic bacterial GGTs including *Escherichia coli* GGT with eukaryotic ones, instead of their nonpathogenic bacterial homologues. This suggests high sequence similarity between pathogens and their hosts. Glutathione hydrolysis by *E. coli* and most of the pathogenic GGTs represents a functional similarity to mammalian GGTs. However, in *Bacillus subtilis* and *Bacillus licheniformis*, the presence of two types of GGT enzymes – the canonical GGT placed in close association to other *Bacillus* GGTs and GGT-like protein YwrD grouped with archaeal and extremophilic GGTs – suggest the horizontal transfer of YwrD protein to the mesophilic *Bacillus* strains from some other archaea or extremophiles. Thus, the diversification of GGT proteins gives intriguing insights into the evolutionary history of this ubiquitously conserved protein. Besides, GGT proteins were also classified into two distinct groups based on the presence or absence of a 12-residue long structural sequence known as lid loop. Accordingly, GGTs from eukaryotes and Gram-negative organisms have been termed as lid-loop positive and GGTs from *Bacillus* species, archaea, and extremophiles as lid-loop negative.

## Physiological Significance of Bacterial GGT

Gamma-glutamyl transpeptidase protein shows widespread conservation, but its physiological role is not very clear yet (Balakrishna and Prabhune, 2014). Mammalian GGTs are involved in glutathione metabolism; on the other hand, prokaryotic GGTs are known to have diverse physiological functions stemming from the fact that the glutathione is not found among all prokaryotes (Table 1).

**TABLE 1:** Classification of bacterial gamma-glutamyl transpeptidases (GGTs) into different physiological groups.

GGT group and associated organisms	Expression and localization	Putative Physiological role
**1. Glutathione degrading GGT** – **mainly present in glutathione producing Gram-negative bacteria**		
*E. coli*	Periplasmic expression	Glutathione utilization as a source of amino acid and nitrogen under nutrient limiting conditions
*Proteus mirabilis*	Localized on the outer cytoplasmic membrane facing the periplasm	
**2. GGT as virulence factor** – **mainly present in pathogenic Gram-negative bacteria**		
*Helicobacter pylori*	Periplasmic expression	Utilization of host’s glutathione and glutamine as a source of glutamate; Inhibition of T-cell proliferation in host
*Campylobacter jejuni*	ND^#^	
*Fransicella tularensis*	ND	Utilization of cytosolic glutathione and γ-glutamyl cysteine peptides present in host cytosol for cysteine acquisition
*Neisseria meningitidis*	Expressed on the inner cytoplasmic membrane facing cytoplasm	Utilization of cytosolic γ-glutamyl cysteine peptides for cysteine acquisition
**3. PGA hydrolyzing GGT** – **mainly present in PGA producing Gram-positive bacteria**		
*Bacillus subtilis (natto)*	Extracellular secretion	Degradation of PGA into glutamate as a source of nitrogen under nutrient-starved conditions
*Bacillus licheniformis*	Extracellular secretion	
Bacillus anthracis*	Membrane-bound expression	Covalent anchorage of PDGA to peptidoglycan layer to maintain capsule integrity; Depolymerization of PDGA inside the cytoplasm

^#^Not determined.*Bacillus anthracis is also a pathogenic strain and GGT is involved in its virulence; however, it has been placed in the third group as it is a Gram-positive *Bacillus* with PDGA as a virulence factor.

Gamma-glutamyl transpeptidase was first reported in some Gram-negative proteobacteria, such as *Proteus mirabilis* and *E. coli*, as a periplasmic protein along with glutathione as the most abundant thiol (Fahey et al., 1978; Nakayama et al., 1984; Suzuki et al., 1986; Masip et al., 2006). In these organisms, deletion or inhibition of the GGT enzyme led to increased extracellular leakage of glutathione (Nakayama et al., 1984; Suzuki et al., 1986). Further studies indicated its possible role in the cleavage of glutathione in periplasmic space to provide cysteine and glycine in Gram-negative bacteria, similar to mammalian GGTs. Another report on *E. coli* GGT indicated its ability to uptake an exogenous supply of γ-glutamyl peptides and glutathione as the source of amino acids (Suzuki et al., 1993). For *Pseudomonas* species, periplasmic localization of GGT has been reported, but its involvement in glutathione metabolism could not be elucidated (Kushwaha and Srivastava, 2014).

In some Gram-negative pathogenic bacteria such as *Helicobacter pylori*, *Fransicella tularensis*, *Neisseria meningitidis*, and *Campylobacter jejuni*, the expression of GGT has been linked to their pathophysiology, and GGT has been ascribed as a formidable virulence factor (Ling, 2013). The pathogenicity mechanism in *H. pylori*, which causes gastritis, gastric ulcers, and cancers in humans, has been studied extensively. *H. pylori* cells colonize gastric mucosa of mammalian gut involving several virulence factors including GGT for the establishment of infection (Ling, 2013; Ricci et al., 2014). During colonization, constitutive periplasmic expression of *H. pylori* GGT (HpGGT) allowed metabolism of extracellular glutathione and glutamine present in the host cytosol as a source of glutamate by *H. pylori* cells (Chevalier et al., 1999; Shibayama et al., 2007). This has been suggested to be the key physiological role played by HpGGT resulting in profuse growth of the pathogen in the gastric mucosa (Gong and Ho, 2004). HpGGT has also been reported to induce apoptosis of gastric epithelial cells under oxidative stress aided by scavenging glutathione from the gastric environment (Shibayama et al., 2003; Gong et al., 2010; Flahou et al., 2011). It has also been demonstrated to have an immunosuppressive effect on the host, as it inhibits the proliferation of T cells resulting in persistent colonization and infection (Schmees et al., 2007). Higher activity of HpGGT from peptic ulcer patients corroborates its clinical relevance as a virulence factor in the diagnosis of gastric-related diseases caused by *H. pylori* (Gong et al., 2010; Franzini et al., 2014). A similar physiological function of GGT was reported in another pathogen *C. jejuni*, closely related to *H. pylori*. GGT from *C. jejuni* assisted in the inhibition of T-cell proliferation, thus promoting persistent colonization of gastric epithelial cells in mammals and avian gut (Barnes et al., 2007; Floch et al., 2014).

*Fransicella tularensis*, a facultative intracellular pathogen, causes tularemia by mainly infecting phagocytic macrophages (Clemens et al., 2004). The role of GGT in its pathogenesis was demonstrated by mutational disruption of *ggt* gene that resulted in drastic defects in its intracellular growth and its replication in macrophage cell lines and caused an attenuated virulence in mice. Here, GGT was reported to be responsible for utilization of glutathione and γ-glutamyl cysteine pools of host as a source of cysteine, essential in intracellular multiplication of pathogen (Alkhuder et al., 2009). In fact, *ggt* deletion mutant of a highly virulent *F. tularensis* SCHU S4 strain has been reported as a potential vaccine candidate; patent was granted in 2013 (United States 8,609,108B2) (Ireland et al., 2011; Le Butt et al., 2013). Another Gram-negative bacteria *N. meningitidis*, the causative agent of a deadly brain disease, meningitis, has also been reported to utilize GGT for cysteine acquisition from an extracellular pool of γ-glutamyl cysteine peptides (Takahashi et al., 2004, 2018). It was located in association with the inner membrane toward the cytoplasmic side, suggesting easy accessibility to extracellular peptides (Takahashi and Watanabe, 2004).

Gamma-glutamyl transpeptidase has been reported in other prokaryotes as well, such as in Gram-positive bacilli including *B. subtilis*, *B. licheniformis*, *Bacillus anthracis* (a pathogen), and *Bacillus amyloliquefaciens*, and in extremophiles such as *Geobacillus thermodentrificans*, *Deinococcus radiodurans*, and *Thermus thermophilus* and archaea like *Picrophilus torridus*. In Gram-positive bacilli, many studies on the putative physiological role of GGTs have ruled out their involvement in glutathione degradation for amino acid utilization, as these bacteria cannot synthesize glutathione (Fahey et al., 1978), although they can hydrolyze it under *in vitro* conditions (Minami et al., 2003b). In another report, when glutathione was supplemented externally as a nitrogen source, the cysteine and glycine auxotrophic mutants of *B. subtilis* could not grow owing to their inability in utilizing glutathione as a source of these amino acids (Xu and Strauch, 1996). However, *B. subtilis* GGT has shown efficacy in cleaving γ-glutamyl bond of glutathione to release cysteinylglycine as a sulfur source instead (Minami et al., 2004). GGT in *Bacillus* species is secreted extracellularly during the onset of the stationary phase, suggesting its role in stationary phase physiology (Xu and Strauch, 1996). Many *Bacillus* species produce poly-γ-glutamic acid (PGA) at the onset of the stationary phase, which is utilized as a nitrogen source during the late stationary phase under nutrient-starved conditions (Ashiuchi and Misono, 2002). The involvement of *B. subtilis (natto)* GGT in the hydrolysis of PGA was investigated for the first time by Kimura et al. (2004). Here, GGT knockout mutant was shown to produce an increased amount of medium-sized PGA fragments (1 × 10^5^ Da) as compared to the wild-type strain, in which both the amount and the size of PGA fragments (5 × 10^3^ Da) decreased considerably. They suggested that the large PGA fragments must have first got hydrolyzed to medium-sized fragments by an endopeptidase YwtD (now known as PgdS) (Suzuki and Tahara, 2003); *B. subtilis* GGT may have acted later as an exopeptidase catalyzing the cleavage of glutamate (both L-and D-forms) residue from amino-terminal of the medium-sized PGA fragments (Kimura et al., 2004). In another report, a double knockout of *pgdS* and *ggt* gene resulted in twofold enhanced production of PGA in *B. subtilis* 168 strain; however, single-gene knockout of *pgdS* or *ggt* did not improve PGA yields (Scoffone et al., 2013). Moreover, among the three mutants, the *pgdS* single mutant produced PGA with the highest molecular weight, which could be attributed to hampered endo-peptidase activity. Contrasting results were obtained in a recent report of *pgdS* and *ggt* knockout mutants of *B. licheniformis* RK14-46 strain. The single *pgdS* gene knockout, as well as the double knockout of *pgdS* and *ggt* gene, resulted in a drastic reduction in PGA production, while the single *ggt* gene knockout improved PGA production due to lesser degradation (Ojima et al., 2019). This suggests that GGT is crucial for PGA hydrolysis by *Bacillus* species, and these GGT-producing Gram-positive bacilli can be classified as PGA degraders.

In addition to GGT, *Bacillus* species like *B. subtilis* and *B. licheniformis* also code for another GGT-like protein named YwrD, expressed intracellularly and shares only 27% sequence similarity with well-described GGTs (Grundy and Henkin, 2001). However, this protein has not been assigned any role yet (Minami et al., 2004).

Another GGT-producing Gram-positive bacterium known to be a deadly pathogen, *B. anthracis*, produces capsular poly-γ-D-glutamic acid (PDGA) as a virulence factor to evade off immune response (Wu et al., 2009). It expresses a virulent protein named capsule depolymerase (CapD), which is located on the cellular surface in association with bacterial envelope (Candela and Fouet, 2005). CapD is considered to be a part of the GGT family, as it, respectively, shares 31 and 27% sequence similarity to *E. coli* and *B. subtilis* GGTs and exhibits similar autoprocessing steps (Uchida et al., 1993; Minami et al., 2004; Wu et al., 2009). CapD has been mainly reported to catalyze the covalent anchoring of PDGA to peptidoglycan for maintaining the integrity of the capsule (Richter et al., 2009). CapD minus *B. anthracis* mutant showed no capsule layer after heat and chemical treatment and was reported to have an attenuated virulence in mice models (Candela and Fouet, 2005). Apart from its anchoring function, CapD has also been suggested to carry out depolymerization of PDGA in the cell cytoplasm (Richter et al., 2009).

Recently, the role of *B. subtilis* GGT (BsGGT) as a novel virulence factor in the pathogenesis of bone resorption similar to mammalian GGT (Niida et al., 2004) has been demonstrated in a cell culture study (Kim et al., 2016). It was reported that the large subunit of BsGGT enhanced osteoclastogenesis activity, independent of enzymatic activity. Further molecular study in the presence of BsGGT large subunit showed that the induction of osteoclastogenesis was related to upregulation of an osteoclast differentiation factor receptor activator of nuclear factor kappa-B ligand (RANKL), which interacted with surface receptors of precursor osteoblast cells and promoted the formation of osteoclast cells. In addition, there was an enhancement in the messenger RNA (mRNA) expression of cyclooxygenase 2 (COX-2). Based on these findings, GGT has been hypothesized to act as a virulence factor in bone destruction, caused by periodontopathic bacteria (Kim et al., 2016).

The physiological significance of GGT in extremophilic microbes has not been elucidated yet. The reason could be the extreme cultural conditions required during cultivation and also the difficulty in the genetic manipulation of such strains.

## Structural and Functional Aspects of Bacterial GGTs

### Structural and Topological Features

**FIGURE 1:**
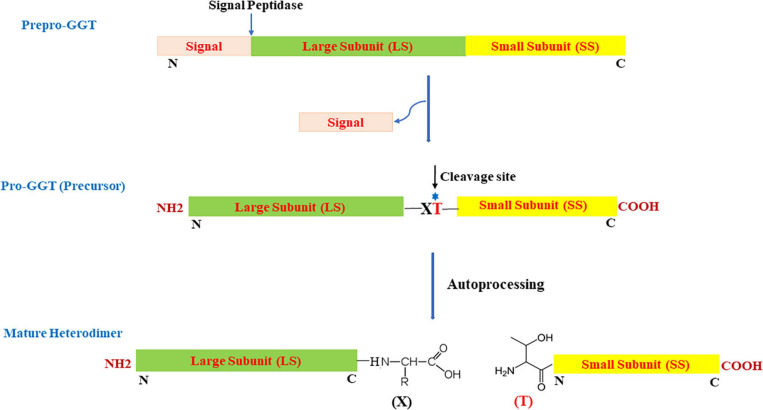
Schematic view of organization and two-step maturation of bacterial gamma-glutamyl transpeptidases (GGTs); the cleavage site represents the site of autoprocessing. T, conserved threonine; X, residue preceding the conserved threonine; N, N-terminus; C, C-terminus.

Most microbial GGTs are synthesized as prepro-GGT with N-terminal signal peptide (Figure 1) sequence for extracellular secretion. After the signal is cleaved, the resulting unique precursor pro-GGT is modified through a self-proteolytic cleavage event termed autoprocessing, forming an active mature heterodimer of large and small subunits (Tate and Meister, 1981; Lo et al., 2007). Autoprocessing involves a conserved nucleophile threonine positioned subcentric inclined toward C-terminal (Balakrishna and Prabhune, 2014). Post autoprocessing, resulting C- and N-terminal, respectively, form large and small subunits; with conserved threonine as the first residue in smaller subunit to serve as a nucleophile for carrying out enzymatic reaction (Inoue et al., 2000; Figure 1).

Primary structure analysis of various microbial GGTs has shown that the intact polypeptide is generally composed of 500 ± 100 amino acid residues (Supplementary Figure 1). GGTs from mesophilic organisms like *E. coli* (EcGGT), *H. pylori* (HpGGT), *Pseudomonas*, and *Bacillus* species contain around 24–35-residue long N-terminal signal peptide, while some extremophilic organisms like *Geobacillus thermodenitrificans* (GtGGT), *Thermus hermophiles* (TtGGT), *D. radiodurans* (DrGGT), etc., lack any signal peptide and are thus intracellular. Primary sequence comparison also reveals >25% sequence similarities among different species, including mammalian GGT from *Homo sapiens* (HsGGT). Further, putative cleavage site for autoprocessing occupied by threonine [T391 in EcGGT; T380 in HpGGT; T399 in *B. licheniformis* GGT (BlGGT)] and two glycine residues (G483/G484 in EcGGT; G481/G482 in BlGGT), constituting oxyanion hole for stabilizing tetrahedral enzyme intermediate during catalysis, is invariably conserved. Large subunit is less conserved and consists of 300–405 residues, while 170–195 amino acid long small subunit is relatively conserved and contains active site residues (Tate and Ross, 1977; Castonguay et al., 2003). These subunits are highly intertwined, mainly by noncovalent hydrophobic interactions and hydrogen bonding (Castellano and Merlino, 2012). The first crystal structure to be elucidated was of *E. coli* GGT (EcGGT) in 2006; in fact, three different structures of EcGGT (PDB IDs: 2DBU, 2D5G, 2DBX) were determined simultaneously (Table 2; Okada et al., 2006). Thereafter, GGT structures from a variety of microbes such as *H. pylori* (HpGGT), *B. subtilis* (BsGGT), *B. licheniformis* (BlGGT), *B. anthracis* (BanGGT; CapD), *Bacillus halodurans* (BhGGT), *Pseudomonas nitoreducens* (PnGGT), and *Thermoplasma acidophilum* (TaGGT) were determined (Boanca et al., 2007; Okada et al., 2007; Williams et al., 2009; Wu et al., 2009; Wada et al., 2010; Ida et al., 2014; Pica et al., 2016; Kumari et al., 2017; Table 2). Although the mammalian GGTs were the first to be described in detail, heavy glycosylation and membrane-bound localization made it difficult to determine their structure; the first crystal structure of human GGT was solved in 2013 (West et al., 2013).

**TABLE 2:** List of crystal structures available for prokaryotic gamma-glutamyl transpeptidase (GGTs).

Enzyme	PDB ID	Resolution	Structural form	GGT molecules in the asymmetric unit
EcGGT	2DBU	1.95	Ligand free form	Heterotetramer – two heterodimeric molecules containing two large and two small subunits
	2DG5	1.60	In complex with hydrolyzed glutathione (substrate)	
	2DBX	1.70	In complex with glutamate (substrate)	
	2DBW	1.80	Acyl-enzyme intermediate	
	2E0Y	2.02	Samarium derivative	
	2E0X	1.95	Monoclinic form (Se-Met GGT)	
	2Z8K	1.65	In complex with acivicin (inhibitor)	
	2Z8J	1.65	In complex with azaserine (inhibitor)	
	2Z8I	2.05	In complex with azaserine in dark	
	2E0W	2.55	T391A mutant	Dimer – two identical precursor molecules
HpGGT	2NQO	1.90	Ligand free form	Heterotetramer – two heterodimeric molecules containing two large and two small subunits
	2QMC*	1.55	T380A mutant in complex with *S*-(nitrobenzyl)glutathione	
	2QM6	1.60	In complex with glutamate	
	3FMN	1.70	In complex with acivicin	
	5BPK	1.49	−	
BsGGT	2V36	1.85	Ligand free form	Heterotetramer – two heterodimeric molecules containing two large and two small subunits
	3A75	1.95	In complex with glutamate	
	3WHS	1.80	In complex with acivicin	Heterodimer – one large and small subunit
	3WHQ	1.58	Soaked in acivicin for 0 min	
	3WHR	1.85	Soaked in acivicin for 3 min	
BlGGT	4OTT	2.98	Ligand free mature form	Heterodimer – one large and small subunit
	4OTU^#^	3.02	In complex with glutamate	
	5XLU	1.45	In complex with acivicin	
	4Y23	2.89	T399A precursor mutant	Monomer – one molecule of precursor
PnGGT	5ZJG	1.70	In complex with Gly–Gly	Heterotetramer – two heterodimeric molecules containing two large and two small subunits
BhGGT (cephalosporin acylase)	2NLZ	2.70	Ligand free form	Heterotetramer – two heterodimeric molecules containing two large and two small subunits
BanGGT (CapD)	3G9K	1.79	Ligand free form	Heterotetramer – two heterodimeric molecules containing two large and two small subunits
	3GA9	2.30	Ligand free form	
TaGGT	2I3O	2.03	Ligand free form	Heterotetramer – two heterodimeric molecules containing two large and two small subunits

*Crystal of an inactive but processed T380A mutant using a bicistronic construct expressing large and small subunit separately.^#^Presence of Mg^2+^ ion crucial for crystal formation.

It has also been demonstrated that in various GGTs, the content of secondary structures formed of α-helices and β-sheets is nearly identical, resulting in similar tertiary and quaternary structural folding (Lin et al., 2014). A highly conserved tetralamellar α/ββ/α fold forms core of the enzyme appearing sandwich-like, made of two tightly packed antiparallel β-sheets, one from each subunit, surrounded by two α-helices (Okada et al., 2006). This fold is characteristic of the N-terminal nucleophile (Ntn) hydrolase superfamily (Brannigan et al., 1995). Most members of this family including penicillin G acylase (Hewitt et al., 2000), cephalosporin acylase (Kim et al., 2000), glycosylasparaginase (Guo et al., 1998), etc., are synthesized as inactive precursors, which undergo autocatalytic processing to form an active mature protein. The activated protein acquires either a nucleophile serine, threonine, or cysteine as its first residue at the newly formed N-terminus with suggested role in catalysis and autoprocessing. Similarly, in case of GGT enzyme, the mechanism of autoprocessing and catalysis also involves a conserved threonine; GGT is therefore considered to be a member of the Ntn hydrolase superfamily.

### Molecular Mechanism of Autoprocessing

**FIGURE 2:**
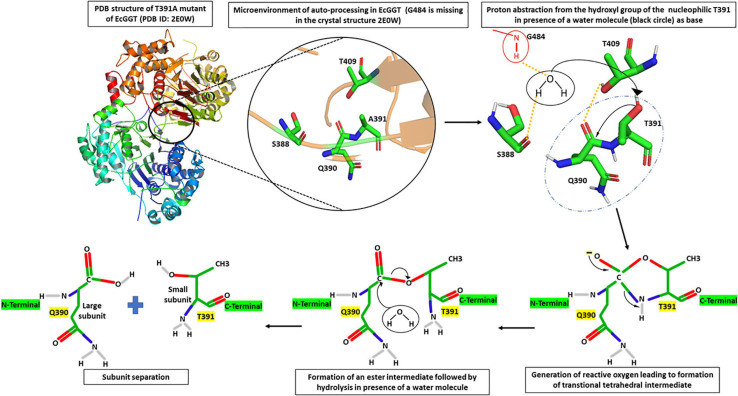
Microenvironment for autocatalytic processing of *Escherichia coli* gamma-glutamyl transpeptidase (EcGGT) and its detailed molecular mechanism. Crystal structure of T391A precursor mutant of EcGGT (PDB ID: 2E0W) has been used for studying autocatalytic environment. A391 in the structure has been replaced by T391 to show hydrogen bonding (yellow dashed lines) and nucleophilic attack (black arrow) by T391 during autoprocessing. A water molecule (black circle) and amide bond of G484 (red circle) have been drawn to show bonding. The structures are prepared using PyMOL2 software.

Through several mutational and crystallography studies, it was demonstrated for the first time in EcGGT that the processing was an autocatalytic event rather than a, earlier hypothesized, protease-dependent occurrence (Kuno et al., 1984). Suzuki and Kumagai (2002) proposed a molecular mechanism of autoprocessing of EcGGT based on its similarity to the Ntn hydrolase superfamily. They suggested that the hydroxyl group of Thr391 (in EcGGT) acts as a nucleophile for proteolytic cleavage of scissile peptide bond with the preceding residue Gln390; the presence of a base abstracts proton from the hydroxyl group of Thr391, generating a reactive oxyanion (Figure 2). The reactive oxygen at gamma position (OG) atom attacks the carbonyl carbon of Gln390 to form a transitional tetrahedral intermediate. The C–N bond later gets cleaved *via* protonation of nucleophile threonine’s amino group, leading to an ester intermediate (N–O acyl shift) which is hydrolyzed further into two subunits with Thr391 as the new N-terminal residue of small subunit (Suzuki and Kumagai, 2002).

Further analysis of the crystal structure of mature *E. coli* GGT (PDB Id: 2DBU), as well as its mutated T391A precursor (PDB ID: 2E0W), provided more mechanistic details of autoprocessing. A water molecule in the close vicinity of Gln390 hydrogen bonded to the hydroxyl group of Ser388 and α-amino group of Gly484 has been reported to enhance nucleophilicity of OG atom of Thr391 that appears crucial in autoprocessing (Figure 2; Okada et al., 2006, 2007). Accordingly, it was reported that substituting conserved threonine in HpGGT (Thr380) and BlGGT (Thr399) with alanine led to complete inhibition of autoprocessing (Boanca et al., 2006; Lyu et al., 2009). Similarly, the cysteine mutant (T399C) of BlGGT remained unprocessed, indicating the importance of the hydroxyl group in cleaving peptide bond (Lyu et al., 2009). Serine mutants of both BlGGT (T399S) and HpGGT (T380S) showed a dramatic decrease in the processing rate; the mutants were, however, processed completely after prolonged incubation. For HpGGT, the role of the methyl group present in the side chain of nucleophile threonine has been suggested for properly positioning the hydroxyl group during the nucleophilic attack (Boanca et al., 2006).

Similar autoprocessing behavior was observed in GtGGT from an extremophilic organism *G. thermodenitrificans* where a homotetrameric precursor is initially produced, which underwent autocatalysis after incubation at 45°C for 48 h (Castellano et al., 2010). The alanine mutant (T353A) of its conserved threonine 353 was also expressed as an inactive unprocessed homotetramer (Castellano et al., 2010).

**FIGURE 3:**
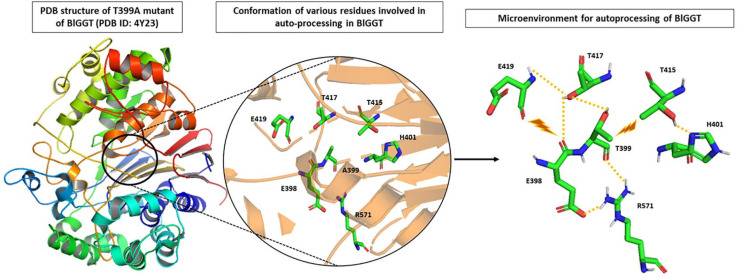
Microenvironment for autocatalytic processing of *Bacillus licheniformis* gamma-glutamyl transpeptidase (BlGGT). Crystal structure of T399A precursor mutant of BlGGT (PDB ID: 4Y23) has been used for studying autocatalytic environment. A399 is replaced by T399 to show hydrogen bonding (yellow dashed lines) and steric hindrance (brown lightning bolt) experienced by T399 for its proper positioning during autoprocessing. The structures are prepared using PyMOL2 software.

Analysis of the recently solved crystal structure of the T399A precursor mimic of BlGGT gave interesting insights; Pica et al. (2016) explored the microenvironment required for autocatalytic processing and proposed a slightly different mechanism of autoprocessing. They suggested that in BlGGT, Thr399 is activated by the OG atom of another Thr417 in lieu of a water molecule as is the case of EcGGT (Figure 3). This leads to a six-membered transition state involving two hydroxyl groups of Thr399 and Thr417 and one carbonyl group of Gln398. Following this, a transient tetrahedral intermediate is formed, and later, hydrolysis of ester bond yields two subunits. Contribution of some other residues, namely, His401, Thr415, Thr417, E419, and Arg571, reported to be important for proper positioning of Gln398 and Thr399, was also assessed by the same group (Figure 3). They generated alanine mutants of each of the five residues and characterized them concerning autoprocessing. Mutants E419A and R571A could process completely, while H401A and T417A showed 60–70% processing in a time-dependent fashion, and T415A mutant persisted as an inactive precursor. Similar maturational defects were observed in EcGGT when the corresponding residue Thr407 (Thr415 in BlGGT) was mutated to aspartate (T407D), lysine (T407K), and serine (T407S) (Lo et al., 2007). In another report from BlGGT, its T417S mutant could process considerably with time, while T417K mutant led to complete blockage of maturation, suggesting the importance of hydroxyl group of Thr417 for proper autoprocessing (Lyu et al., 2009). In HpGGT, mutation of the same threonine residue at position 398 to serine (T398S) and alanine (T398A) resulted in comparable processing rates with respect to native HpGGT (Boanca et al., 2007). Thus, despite being highly conserved among microbial as well as mammalian GGTs, the role of this threonine residue in autoprocessing is still controversial.

The role of residues present in the immediate vicinity of the cleavage site during autoprocessing was also explored. The residue preceding conserved nucleophile threonine was variable among microbial GGTs (Supplementary Figure 1). After autoprocessing, it acquired the last position of the newly formed C-terminus of the large subunit and was occupied by Gln390 in EcGGT and Glu398 in BlGGT. Analysis of four mutants, *viz* E398A, E398D, E398R, and E398Q of BlGGT, suggested that the C-terminal of the large subunit was indeed critical for autoprocessing of BlGGT (Chi et al., 2012). Among these, in three mutants, except E398Q, autoprocessing was hampered, which did not improve even after prolonged *in vitro* incubation. On the contrary, in EcGGT, replacement of the corresponding residue Q390 with alanine (Q390A) did not affect autoprocessing, indicating the insignificance of the large subunit C-terminus of EcGGT in autoprocessing (Hashimoto et al., 1995). The obtained contradictory results also possibly explain the low conservation of this residue among prokaryotic GGTs. Later, Chi et al. (2014) demonstrated the role of a 14-residue long extra sequence, exclusively present in *Bacillus* GGTs at the C-terminus of the large subunit, in autoprocessing, structural stability, and catalysis of BlGGT. They constructed six progressive deletion mutants within the extra sequence region with altered autoprocessing capabilities and variable catalytic activities (Chi et al., 2014). Further, the N-terminal of the small subunit, constituting a highly conserved TTH motif, was also investigated for its role in autoprocessing. The role of the first threonine residue, constituting the conserved nucleophile, in autoprocessing has been established well for many bacterial GGTs (Suzuki and Kumagai, 2002; Pica et al., 2012, 2016). In EcGGT, replacement of the second and third residue, respectively, with alanine (T392A) and glycine (H393G) impaired autoprocessing, suggesting that the small subunit N-terminus is more crucial than the large subunit C-terminus during autoprocessing (Hashimoto et al., 1995).

Apart from this, N- and C-termini of the intact polypeptide chain (prepro-GGT) of bacterial GGTs were also studied for their importance in autoprocessing. Truncation in the N-terminal region, including the signal peptide, of EcGGT and BlGGT demonstrated that signal peptide is critical for proper enzyme folding and processing (Lin et al., 2008; Lo et al., 2008). For EcGGT, truncation beyond 17 amino acids was found detrimental for maturation, indicating the crucial role of the last nine amino acids of the signal peptide in functional expression of the enzyme (Lo et al., 2008), while in BlGGT, all the truncated mutants except the native enzyme resulted in partial or complete loss of processing, emphasizing again on the importance of signal peptide in the enzyme’s folding and maturation (Lin et al., 2008). This is contradictory to the reports showing functional heterologous expression, with efficient autoprocessing, of EcGGT and BlGGT that lack signal peptide sequence (Lin et al., 2006; Yao et al., 2006). Similarly, N-terminal truncation in BpGGT up to 95 residues did not affect autoprocessing, but functional activity was lost with truncation beyond 63 residues (Murty et al., 2012). The importance of the C-terminus in autoprocessing has also been elucidated and was first demonstrated in EcGGT. Two arginyl residues present at the C-terminus of EcGGT were mutated with alanine (R513A) and glycine (R571G), resulting in a complete blockage of autocatalytic processing (Hashimoto et al., 1992). Further in HpGGT, the formation of salt bridges among four highly conserved residues, *viz* Glu515/Arg547 and Arg502/Asp562 at C-terminus, suggested their importance in enzyme processing. Disrupting salt bridges by mutating residue Glu515 (E515Q) and Arg502 (R502L) led to impaired autoprocessing and activity (Williams et al., 2009). C-terminal truncation in BlGGT revealed that deletion beyond V576, at ninth position from C-terminus, was detrimental for autoprocessing and activity (Chang et al., 2010).

### Effect of Autoprocessing on Structure and Function of GGT

Structural analysis of EcGGT and HpGGT crystal structures showed that the new termini formed post autoprocessing are approximately 35 Å apart, which is quite distant, suggesting significant conformational changes during autoprocessing (Okada et al., 2006; Morrow et al., 2007). Further, comparing the overall structure of the mature and T391A precursor of EcGGT demonstrated that backbone atoms in the core regions of both the proteins remain unchanged, while significant conformational changes occurred proximal to the active site (Okada et al., 2007). Particularly after autoprocessing, the large subunit C-terminus (denoted as P-segment) flips out and is replaced by a flexible lid loop located in the smaller subunit. Adoption of an extended conformation by the P-segment has been suggested significant in forming binding pocket. However, these conformational changes do not occur in all microbial GGTs owing to the absence of lid loop as seen in *Bacillus* species. Analysis of the mature structures of BsGGT and BlGGT demonstrated that the C-terminus of large subunit possesses an extra sequence region located close to the N-terminus of the small subunit, suggesting no significant conformational changes upon autoprocessing (Wada et al., 2010; Lin et al., 2014). The extra sequence in BlGGT has been reported as important for autoprocessing and enzyme activity (Chi et al., 2014). Apart from structural implications, the correlation of autoprocessing with catalytic activity has also been well documented. Analysis of *in vitro* processing of native and mutant precursors of EcGGT and BlGGT, as a function of time, suggests that the extent of autoprocessing correlates well with increased enzyme activity (Suzuki and Kumagai, 2002; Lin et al., 2008). In GGTs like EcGGT, HpGGT, and BpGGT, uncoupling of autoprocessing and enzymatic activity has been attempted. Hashimoto et al. (1995) demonstrated that the coexpression of large and small subunit (using separate expression plasmid for each subunit) of EcGGT could result in reconstitution of two subunits under both *in vivo* and *in vitro* conditions. The retained activity was very less, indicating that only few molecules of the two subunits could fold properly and give rise to an active form (Hashimoto et al., 1995). In contrast, for HpGGT and BpGGT, coexpression of both the subunits in pET-DUET vector resulted in a fully active heterodimeric enzyme with comparable and enhanced activities (Boanca et al., 2006; Murty et al., 2012). This indicated that different bacterial GGTs can adopt different conformations; use of different expression systems can also effect both folding and active conformation of GGT, as uncoupling autoprocessing from enzymatic activity was successful for HpGGT and BpGGT but not for EcGGT.

### Catalysis Mediated via Nucleophile Threonine

**FIGURE 4:**
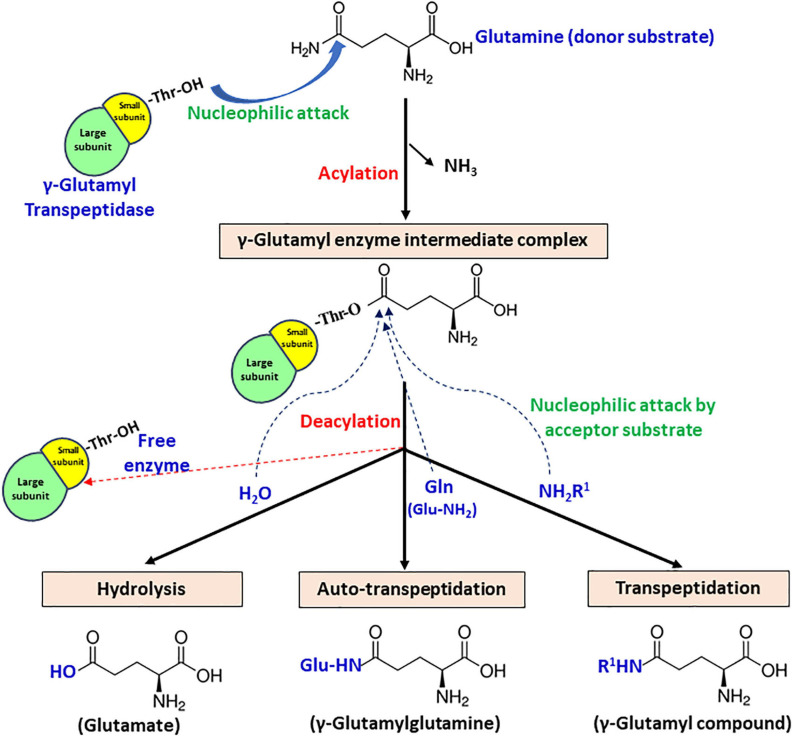
Schematic representation of the proposed catalytic mechanism of gamma-glutamyl transpeptidase (GGT) with glutamine as the γ-glutamyl moiety donor.

Gamma-glutamyl transpeptidase enzyme catalyzes a two-step reaction, involving cleavage of γ-glutamyl bond present in γ-glutamyl compounds like glutathione and glutamine subsequently, followed by transfer of γ-glutamyl moiety to a water molecule or another amino acid or short peptide (Tate and Meister, 1981). The first step is termed acylation, in which donor substrate donates γ-glutamyl moiety to the enzyme, forming an acyl-enzyme intermediate. In the second step termed deacylation, an acceptor substrate accepts γ-glutamyl moiety to form final end product (Figure 4). Based on the end product formation, GGT mediates three types of reactions.

1. Hydrolysis – acceptor is a water molecule, with end-product glutamate.
2. Transpeptidation – acceptor is an amine group containing compound that can be any amino acid or short peptide, and the end-product formed is a γ-glutamyl compound.
3. Autotranspeptidation – acceptor is the donor molecule itself, and the end-product is γ-glutamylated donor.

The formation of an acyl-enzyme intermediate during catalysis has been substantiated by both kinetic and crystallographic studies. Stop-flow kinetics performed under presteady-state conditions using rat kidney GGT revealed a biphasic pattern of enzyme catalysis, confirming its ping-pong mechanism (Keillor et al., 2004). Based on the observation, it has been interpreted that the acylation step is faster, while the deacylation step is rate limiting (Keillor et al., 2005). For bacterial GGTs, further examination of the X-ray crystal structure of EcGGT soaked in glutathione for varying time intervals also demonstrated the formation of γ-glutamyl enzyme intermediate, indicating that the second step is much slower than the first (Okada et al., 2006). Further analysis of EcGGT in complex with substrate analogs, acivicin and azaserine, revealed that they formed a tetrahedral adduct (structurally analogous to transient acyl-enzyme intermediate) with the enzyme (Wada et al., 2008).

Gamma-glutamyl transpeptidase-mediated reactions are known to be catalyzed by nucleophilic attack on the donor substrate. The N-terminal threonine residue of small subunit responsible for autoprocessing has also been reported as the relevant nucleophile in enzyme catalysis (Inoue et al., 2000). Inhibition of GGT activity by treatment with certain inhibitors like 6-diazo-5-oxo-L-norleucine (DON) and serine–borate complex suggested the involvement of the hydroxyl group of either a serine or a threonine residue (Tate and Meister, 1977, 1978). However, Inoue et al. (2000) were the first group to identify that the conserved Thr391 is indeed the catalytic nucleophile for EcGGT. They employed mechanism-based affinity labeling to modify the putative active site of GGT using 2-amino-4-(fluorophosphono) butanoic acid (1), a γ-phosphonic acid monofluoride derivative of glutamic acid. The compound phosphonylated the putative catalytic nucleophile, and subsequent liquid chromatography/mass spectrometry (LC/MS) and MS/MS analysis revealed that the small subunit N-terminal threonine is the catalytic nucleophile. Moreover, the structure of EcGGT in complex with glutathione clearly illustrates the formation of a covalent bond between the OG atom of Thr391 and carbonyl carbon of γ-glutamyl moiety of glutathione (Okada et al., 2006).

Analysis of the crystal structure of HpGGT also demonstrated the role of a second threonine (T398) residue in increasing nucleophilicity of the catalytic threonine (T380) (Boanca et al., 2007). It has been reported to be highly conserved among bacterial GGTs and suggested to interact with the hydroxyl group of the catalytic threonine, resulting in the formation of a TT dyad. Besides, the nucleophilicity of catalytic threonine has also been proposed to increase through interaction with its own α-amino group, which can act as a base to activate catalytic nucleophile threonine (Michalska et al., 2005; Boanca et al., 2007). The contribution of T398 in the activation of T380 in HpGGT was investigated by site-directed mutagenesis. Replacement of T398 with alanine (T398A) and serine (T398S) resulted in a complete and nearly fivefold reduction of enzymatic activity, respectively, with no maturational defects, suggesting the importance of TT dyad in efficient catalysis. A plausible explanation given for the same was the presence of a methyl group in T398, likely required for precise positioning of its side chain hydroxyl group for interaction with catalytic threonine (Boanca et al., 2007). Similarly, the replacement of the corresponding second threonine residue T417 with lysine, serine, and alanine in BlGGT also resulted in a dramatic decrease in activity as well as autoprocessing rate, indicating its functional significance in both autoprocessing and catalysis (Lyu et al., 2009).

### Substrate Binding Pocket

**FIGURE 5:**
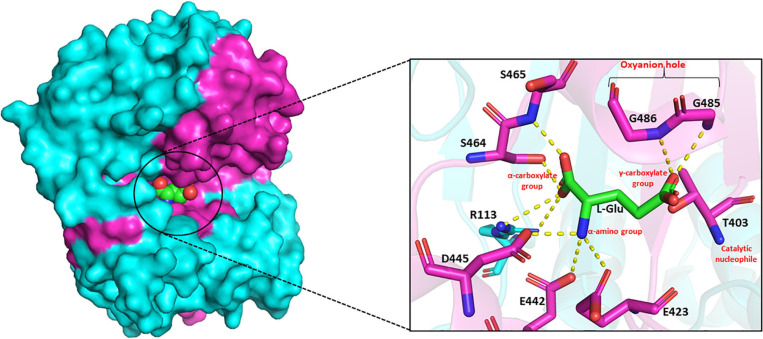
Side view of surface drawing of *Bacillus subtilis* gamma-glutamyl transpeptidase (BsGGT) (PDB ID: 3A75) showing the binding pocket groove and interaction of L-glutamic acid (L-Glu) with active site residues of BsGGT. Cyan color highlights large subunit, pink color highlights small subunit, and L-Glu is represented as a sphere in the surface drawing. Hydrogen bonding is represented as yellow dashed lines. The structures are prepared using PyMOL2 software.

The substrate-binding pocket of GGT can be divided into two parts – the donor binding site and the acceptor binding site. The donor binding site interacts with γ-glutamyl moiety in the donor molecule, while the presence of a discrete acceptor binding site is still ambiguous. In mammalian GGTs, these sites have been reported to be largely overlapping (Thompson and Meister, 1977). However, in bacterial GGTs, the donor binding site has been determined to be highly conserved, while the acceptor binding site is reported highly variable. Analysis of the crystal structures of EcGGT, HpGGT, BsGGT, and BlGGT in complex with glutamate demonstrated that the catalytic pocket is enclosed within the shallow groove at the interface of both large and small subunits (Okada et al., 2006; Morrow et al., 2007; Wada et al., 2010; Lin et al., 2014). The binding pocket resides the catalytic threonine at the bottom of the groove on one of the central β-sheets, and it further extends into the enzyme, appearing as a finger-like projection. The γ-glutamyl moiety of the donor molecule is bound to the active site at the bottom of the pocket by an extensive network of hydrogen bonds and salt bridges formed mainly with the residues present in the small subunit (Okada et al., 2006). In BsGGT, the α-carboxylate group of the bound glutamate interacts with Arg113, Ser464, and Ser465, and the α-amino group interacts with Glu423, Glu442, and Asp445 (Figure 5; Wada et al., 2010). The glutamate carbonyl carbon is covalently bonded to the OG atom of threonine, while the carbonyl oxygen is hydrogen bonded to the main chain amino group of two glycine residues, Gly485 and Gly486. Arg113 is reported to be the only residue from, respectively, the large subunit involved in binding pocket formation. Residues forming the binding-pocket of mesophilic GGTs are mostly conserved, except differences of one or two residues, suggesting rigidity of the binding pocket: in EcGGT, Asn411 and Gln430 while Glu423 and Glu442 in BsGGT. On the contrary, GGTs from extremophiles exhibit variability in their catalytic residues with respect to their mesophilic counterparts as has been revealed by multiple sequence alignment (Supplementary Figure 1). For example, the highly conserved arginine residue (R107 in BlGGT; R113 in BsGGT; R114 in EcGGT), present in the large subunit of mesophilic GGTs, is replaced by serine (S68 in PtGGT and S85 in GtGGT) in extremophiles. Likewise, the two consecutive serine residues (Ser462/Ser463 in EcGGT) of small subunit are replaced by histidine and threonine (414His/415Thr in GtGGT) in GGTs from extremophiles. The different nature of these residues is suggested to be responsible for the differences in catalytic properties of mesophilic and extremophilic GGTs (Castellano et al., 2011).

Recent crystallographic studies on L-Glu-bound BlGGT demonstrated that its active site channel constitutes two extensive pockets at the intersubunit interface lined mainly by hydrophobic residues. In addition, substrate binding resulted in significant structural changes in BlGGT; particularly, there is a reordering of six residues at the C-terminus of the large subunit with suggested role in enzyme catalysis (Lin et al., 2014).

As GGT catalysis follows a ping-pong mechanism, it is assumed that it sequentially binds donor and acceptor molecules. It has been suggested that the acceptor molecule occupies the same site where initially the leaving group of the donor molecule binds, but nothing has been conclusively proven yet (Taniguchi and Ikeda, 1998). Hu et al. (2012) threw some light on the acceptor binding residues and their mode of interaction by computational and mutational studies performed using CapD protein from *B. anthracis* and HsGGT (*Homo sapiens*). Analysis of the structures of CapD and HsGGT suggested that the putative acceptor binding site is proximal to the donor binding site, located in the deep groove, and is highly variable and flexible as compared to the conserved and rigid donor binding site. Further, the putative acceptor binding site of HsGGT has been suggested to be formed by some polar residues like Asp46, Asn79, His81, Ser82, Tyr403, Gln476, and Lys562, while in CapD, Arg423 and Arg520 are substituted in place of Gln476 and Lys562. Both the residues Arg423 and Arg520 have been suggested to play a role in acceptor binding and imparting broader stereospecificity to CapD in accepting both L- and D-forms of amino acids as acceptors.

Interestingly, *Pseudomonas nitroreducens* GGT (PnGGT) exhibits higher hydrolytic activity than transpeptidase activity despite high sequence similarity with EcGGT (Imaoka et al., 2010). Recent crystallographic studies on PnGGT in complex with Gly–Gly acceptor demonstrated that the binding mode of Gly–Gly in the active site is probably the reason for its reduced activity as an acceptor (Hibi et al., 2019). The terminal amino group of Gly–Gly is oriented opposite to the nucleophilic active center. Moreover, tight packaging of three aromatic residues Trp385, Phe417, and Trp525 around the Gly–Gly binding pocket has been suggested to be advantageous for its binding; side chains of these residues are involved in the recognition of acceptor substrate. Phe417 is located in the lid loop, while Trp385 and Trp525 have been suggested to form side walls of the putative acceptor binding site. However, the hydrophobic pocket formed by the three aromatic residues has been suggested to shield the catalytic nucleophile from bulk solvent and activating hydrolysis. The functional significance of the putative acceptor site of PnGGT including Trp385, Phe417, and Trp525 has also been explored by mutational studies. Substitution of three aromatic residues with threonine (W385T), tyrosine (F417Y), and alanine (W525A), present at the corresponding positions in EcGGT, resulted in enhanced transpeptidase activity for all three mutants, while a 5–14% decrease in hydrolytic activity was observed. Likewise, in another report from *Picrophilus torridus* GGT (PtGGT), the replacement of an aromatic residue tyrosine at position 327 by asparagine (corresponding residue in EcGGT) introduced significant transpeptidase activity in PtGGT, while the native enzyme exhibited only hydrolytic activity (Rajput et al., 2013). Comparative docking of the acceptor ligand Gly–Gly in the structural model of PtGGT and its Y327N mutant identified some residues with suggested importance in acceptor recognition and binding. The acceptor Gly–Gly interacted with five residues (L87, E90, Y305, N327, S348) and perfectly docked in the binding pocket of Y327N.

### Importance of Lid Loop in Catalysis

**FIGURE 6:**
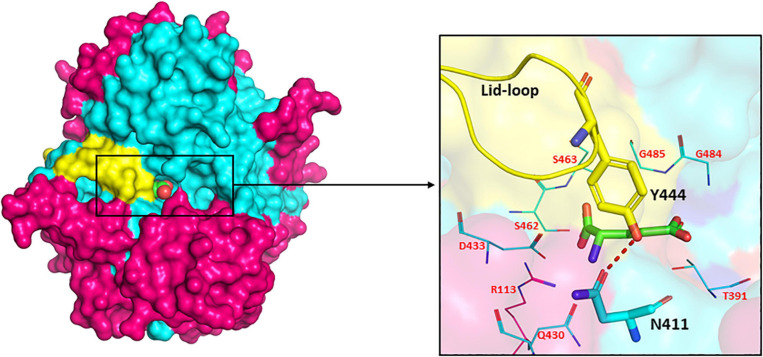
Surface drawing of *Escherichia coli* gamma-glutamyl transpeptidase (EcGGT) (PDB ID: 2DBX) showing the arrangement of lid-loop around substrate-binding pocket. Pink color highlights large subunit, cyan color highlights small subunit, and L-Glu is represented as a sphere in the surface drawing. Hydrogen bonding between residues Asn411 and Tyr444 is shown by the red dotted line. The active site residues surrounding the bound substrate L-Glu are pictorially shown as lines. The structures are prepared using PyMOL2 software.

The presence of a unique lid-loop region in the small subunit of GGT of different bacterial and mammalian homologues as shown in multiple sequence alignment (Supplementary Figure 1) has structural and functional significance. Analysis of substrate-bound crystal structures of EcGGT and HpGGT demonstrated that the otherwise flexible lid loop acquires a well-defined position after binding of the substrate and extends over the binding site pocket, shielding it from the solvent molecule (Okada et al., 2006). The lid-loop spanning segment from Pro438 to Gly449 in EcGGT contains an aromatic residue tyrosine (Try444), which has been reported to form a hydrogen bond with conserved residue Asn411 by its side chain hydroxyl group (Figure 6). The hydrogen bond has been suggested to act as a gate, restricting entry of the solvent molecule into the active site and also regulating access of the substrate to the active site cleft that results in a rigid structural conformation around the binding pocket. A similar hydrogen bond has been reported between Tyr433 of the lid loop and Asn400 in HpGGT (Morrow et al., 2007). The shielding likely occludes water molecules from entering the active site, facilitating transpeptidation. However, human GGT exhibits higher transpeptidase activity, likely associated with higher mobility of its lid loop allowing rapid switch of its structural conformation from open to close resulting in faster product release (Hu et al., 2012). Analysis of recently determined crystal structure of human GGT also confirms high mobility of the lid loop (West et al., 2013). The probable explanation ascribed is the replacement of tyrosine with phenylalanine (Phe444), causing a lack of stabilizing hydrogen bond. In HpGGT, substitution of Try433 with Phe (Y433F) had no effect on the catalytic activity, but mutation with alanine (Y433A) resulted in a dramatic decrease in activity; substrate binding was, however, not affected (Morrow et al., 2007).

Several microbial GGTs including BsGGT, BlGGT, GtGGT, TaGGT, PtGGT, DnGGT, and TtGGT lack the lid-loop segment. Structural analysis of BsGGT and BlGGT demonstrated that their binding pocket is solvent exposed, as they lack lid loop or any of that sort to cover the active site. They are thus suggested to have an open active site cleft to accommodate hydrolysis of large polymeric substrates like PGA (Wada et al., 2010; Hu et al., 2012; Lin et al., 2014). Based on this correlation between the absence of lid loop and hydrolysis of higher molecular weight substrates, it has been proposed that lid loop containing enzymes would not be able to utilize polymeric substrates due to closed catalytic pocket. In an interesting study by Calvio et al. (2018), the role of lid loop in substrate selection was investigated where they constructed a mutant BsGGT by inserting the lid loop from EcGGT. On comparing the activities of EcGGT (lid loop), BsGGT (no lid loop), and mutant BsGGT (lid loop), they concluded that entry of substrate into the active site was regulated by the lid loop depending on molecular weight. Both EcGGT and mutant BsGGT could efficiently utilize glutamine than bulky PGA. Moreover, the presence of the lid loop in mutant BsGGT enhanced its transpeptidation activity as compared to EcGGT and BsGGT (Calvio et al., 2018).

## Mutations in Binding Pocket

Site-directed mutagenesis of the active site residues of microbial GGTs has contributed significantly in determining their functional role and also in the development of catalytically efficient variants (Table 3). As already mentioned, the only residue of the large subunit reported to participate in catalysis is the conserved arginine (Arg114 in EcGGT, Arg113 in BsGGT, Arg109 in BlGGT). This residue has been reported to form a weak interaction with the α-carboxylate group of donor substrate (Ikeda et al., 1993; Okada et al., 2006). However, the role of this residue has been elucidated in substrate recognition and binding rather than direct involvement in catalysis. In BlGGT, replacement of this arginine with another positively charged residue lysine (R109K) resulted in significant enhancement of activity (Bindal et al., 2017), whereas in EcGGT (R114K) and BsGGT (R113K), similar mutation resulted in 93 and 22% residual activities, respectively (Minami et al., 2003a; Ong et al., 2008). Substitutions with amino acids of different functional nature such as Leu, Met, Asp, Glu, Phe, etc., were detrimental (Table 3). R109M and R109L mutants of BlGGT exhibited only hydrolytic activity (Bindal et al., 2017).

**TABLE 3:** List of mutations performed in conserved and catalytically important residues of bacterial gamma-glutamyl transpeptidases (GGTs) and their effect on autoprocessing and activity.

Residue	Mutation	Effect	References	Suggested functional role
Arg113 in large subunit of EcGGT*				
Arg114 (EcGGT)	R114K	93% activity retained	Ong et al., 2008	Substrate recognition and binding
	R114L	No detectable activity		
	R114D	No detectable activity		
Arg113 (BsGGT)	R113K	Reduced activity by 78%	Minami et al., 2003a	
Arg109 (BlGGT)	R109K	2-fold enhancement in activity	Bindal et al., 2017	
	R109S	85% activity retained		
	R109L	Abolished transpeptidase activity; 5% hydrolytic activity retained		
	R109M	Abolished transpeptidase activity; 4% hydrolytic activity retained		
	R109F	No detectable activity		
	R109E	No detectable activity		
**Thr391 at small subunit N-terminal of EcGGT**				
Thr391 (EcGGT)	T391A	No autoprocessing and detectable activity	Suzuki and Kumagai, 2002	Catalytic nucleophile crucial for both autoprocessing and activity
	T391S	Impaired autoprocessing and activity (values not mentioned)		
	T391C	Impaired autoprocessing and activity (values not mentioned)		
Thr380 (HpGGT)	T380A	No autoprocessing and detectable activity	Boanca et al., 2006	
	T380S	Impaired autoprocessing; reduced activity by 92%		
Thr399 (BlGGT)	T399A	No autoprocessing and detectable activity	Lyu et al., 2009	
	T399S	Impaired autoprocessing; reduced activity by 89%		
	T399C	No autoprocessing and detectable activity		
Thr353 (GtGGT)	T353A	No autoprocessing; retained some activity (values not mentioned)	Castellano et al., 2010	
**Asp433 in small subunit of EcGGT**				
Asp445 (BsGGT)	D445A	Abolished transpeptidase activity; 40% hydrolytic activity retained	Minami et al., 2003a	Substrate binding and its affinity
	D445E	Reduced activity by 86%		
	D445N	Reduced activity by 90%; cephalosporin acylase activity increased by 55-fold		
	D445Y	Reduced activity by 91%		
	E423Y/E442Q/D445N	Cephalosporin acylase activity enhanced by 963-fold	Suzuki et al., 2010	
Asp433 (EcGGT)	D433N	Abolished transpeptidase activity 82% hydrolytic activity retained; introduced cephalosporin acylase activity	Suzuki et al., 2004c	
	D433N/Y444A/G484A	Cephalosporin activity enhanced by 50-fold	Yamada et al., 2008	
**Gly484 and Gly485 in small subunit of EcGGT**				
Gly481 (BlGGT)^#^	G481A	80% autoprocessed with 78% activity retained	Chi et al., 2018	Stabilization of oxyanion hole during catalysis; crucial for both autoprocessing and catalysis
	G481R	Complete loss of autoprocessing and activity		
	G481E	60% autoprocessed with 13% activity retained		
Gly482 (BlGGT)^#^	G482A	95% processed; retained 70% activity		
	G482R	20% processed; no detectable activity		
	G482E	80% processed; significant loss in activity (4% left)		
**PLSSMXP motif in small subunit of EcGGT (Pro460, Leu461, Ser462, Ser463, Met464, Pro466)**				
P458 (BlGGT)	P458A	25% increase in activity	Chi et al., 2019b	Crucial for both autoprocessing and catalysis
L459 (BlGGT)	L459A	No effect on activity		
S460 (BlGGT)	S460A	2.1-fold enhancement in activity		
S461 (BlGGT)	S461A	2.4-fold enhancement in activity		
S463 (EcGGT)	S463T	Reduced activity by 40%	Hsu et al., 2009	
	S463K	No autoprocessing and detectable activity		
	S463D	No autoprocessing and detectable activity		
M462 (BlGGT)	M462A	37% increase in activity	Chi et al., 2019b	
M464 (EcGGT)	M464E	No autoprocessing and detectable activity	Lo et al., 2007	
	M464K	No autoprocessing and detectable activity		
	M464L	Reduced activity by 50%		
P464 (BlGGT)	P464A	Reduced activity by 53%	Chi et al., 2019b	
Deletion mutants (BlGGT)	△M462	No autoprocessing and detectable activity	Chi et al., 2019b	
	△*S*460-M462			
	△*S*461-M462			
	△*P*464			
**Asp452 residue in small subunit of EcGGT**				
Asn450 (BlGGT)**^†^**	N450Q	41% increase in transpeptidase activity	Lin et al., 2016	Substrate binding and catalysis
	N450A	3.5-fold enhancement in transpeptidase activity		
	N450D	3.6-fold enhancement in transpeptidase activity		
	N450K	Reduced activity by 27%		

*The residues have been numbered according to their corresponding position in *E. coli* GGT for ease in reading the table. The activities mentioned in the table are transpeptidase activities measured in presence of both donor and acceptor; hydrolytic activity wherever mentioned is measured only with donor without the addition of any acceptor substrate.^#^The transpeptidase activity was recorded after an incubation of 21 days at 4°C.^†^Transpeptidase and hydrolytic activities were recorded after 10 days of incubation at 4°C.

Another active site residue of the small subunit (Asp445 in BsGGT) reported to interact with the α-amino group of donor substrate has also been suggested to have a putative role in substrate binding (Minami et al., 2003a). Replacement of Asp445 by alanine (D445A) resulted in a hydrolytic variant with 40% retained activity and complete abolishment of transpeptidase activity with respect to native BsGGT (Minami et al., 2003a). Likewise, substitution of the corresponding residue Asp433 with asparagine (D433N) in EcGGT abolished its transpeptidase activity, and the hydrolytic variant evolved retained almost 82% activity (Suzuki et al., 2004c). D433N mutant could catalyze deacylation of glutaryl-7-aminocephalosporanicacid (GL-7-ACA) producing 7-aminocephalosporanic acid, a starting material for the synthesis of semisynthetic cephalosporins. GGTs share 30% sequence homology with class IV cephalosporin acylases (CAs) that synthesize semisynthetic cephalosporins. The conserved Asp433 residue of EcGGT is replaced by asparagine in class IV CAs; point mutation (D433N) introduced CA activity in EcGGT, although the acylase activity was quite low; introduction of two random mutations of Y444A and G484A to D433N resulted in up to 50-fold increase in catalytic efficiency for GL-7-ACA substrate (Yamada et al., 2008). Similarly, a triple mutant E423Y/E442Q/D445N of BsGGT was also developed with enhanced acylase activity (Suzuki et al., 2010).

The functional role of two fully conserved tandem glycine repeats reported to form the oxyanion hole has also been explored (Chi et al., 2018). In BlGGT, substituting residues Gly481 and Gly482, respectively, with arginine (G481R) and lysine (G481K) led to impaired autoprocessing as well as catalytic activity, while alanine substitution was not detrimental (Table 3). It has been suggested that proper positioning of the glycine residues is critical for both autoprocessing and catalysis (Chi et al., 2018).

Recently, a highly conserved ^458^PLSSMXP^464^ motif present in the small subunit of BlGGT containing the two catalytic serine residues has been studied by deletion and alanine scanning mutagenesis (Chi et al., 2019b). All the deletion mutants showed complete loss of processing and activity. However, alanine substituent of four residues Pro458 (P458A), Ser460 (S460A), Ser461 (S461A), and Met462 (M462A) exhibited a significant increase in catalytic activity with no maturational defects (Table 3). Interestingly, S460A and S461A displayed about twofold enhancement in activity; the role of catalytic Ser463 in EcGGT corresponding to Ser461 in BlGGT was also investigated by mutating it to aspartate (S463D), lysine (S463K), and threonine (S463T). Variants S463D and S463K of EcGGT showed no detectable activities with considerable maturational defects (Hsu et al., 2009), while S463T mutant displayed 40% loss in activity without affecting autoprocessing, indicating that presence of hydroxyl group is required for autoprocessing and addition of charged residues at this position is detrimental for both processing and activity. The same phenomenon was observed for Met464 residue in EcGGT (Met462 in BlGGT) wherein M464E and M464K were maturationally blocked mutants, while M464L exhibited no loss in autoprocessing but had a 50% loss in activity (Lo et al., 2007).

Analysis of the L-Glu-bound crystal structure of BlGGT (PDB ID: 4OTU) suggested importance for another highly conserved residue, Asn450, located close to the binding pocket, in proper substrate orientation (Lin et al., 2016). Replacement of Asn450 by glutamine (N450Q), aspartate (N450D), and alanine (N450A) showed a significant increase in transpeptidase activity up to 3.6-fold enhancement and minor maturational defects, suggesting significant contribution of this residue in substrate binding and catalysis.

Based on mutations in catalytically conserved residues of bacterial GGTs, it was observed that contradictory results were obtained for similar mutations in different bacterial GGTs concerning autoprocessing and catalytic activities (Table 3). The possible reason for the same may be attributed to minor differences in the autoprocessing microenvironments and active structural conformations of different bacterial GGTs. For example, the presence of the lid loop in EcGGT can also influence its catalysis in comparison to *Bacillus* GGTs lacking the lid-loop region.

## Physicochemical Properties of Bacterial GGTs

Wild strains like *E. coli* and *Bacillus* species produced very low titers of GGT, as it is a stress-related enzyme (Suzuki et al., 1987; Minami et al., 2003b). Further, cultivation of extremophilic organisms like *G. thermodenitrificans*, *D. radiodurans*, etc., is rather difficult; therefore, bacterial GGTs have been largely expressed heterologously in *E. coli* and *B. subtilis* via conventional methods for their biochemical and biophysical characterization (Table 4).

**TABLE 4:** Heterologous expression of prokaryotic gamma-glutamyl transpeptidases (GGTs).

S. No.	Enzyme Source	Expression Host	Purification	Specific activity (U/mg)	Remarks	References
1.	*E. coli* K-12	GGT deficient *E. coli* K-12	Two-step; ammonium sulfate precipitation and chromatofocusing	3.0	37-fold enhanced expression; 4.4-fold purification with 42% yield	Suzuki et al., 1988
2.	*E. coli* Novablue	*E. coli* M15	One-step; Ni-NTA affinity chromatography utilizing N-terminal His_6_ tag	4.25	32.7-fold purification with 83% yield	Yao et al., 2006
3.	*Bacillus licheniformis* ATCC 27811	*E. coli* M15	One-step; Ni-NTA affinity chromatography utilizing N-terminal His_6_ tag	185. 6	29-fold purification with 26 mg/L yield	Lin et al., 2006
4.	*Geobacillus thermodenitrificans* NG80-2	*E. coli* BL21 (DE3)	One-step; Ni-NTA affinity chromatography utilizing C-terminal His_6_ tag	0.36 (hydrolytic)	10 mg/L purification yield; no transpeptidase activity	Castellano et al., 2010
5.	*Pseudomonas nitroreducens* IFO12694	*E. coli* Rosetta-gami B (DE3)	Two-step; DEAE cellulofine and Butyl FF column chromatography	30.2 (hydrolytic); 2.98 (transpeptidase)	70-fold enhanced expression; high hydrolytic activity	Imaoka et al., 2010
6.	*Thermus thermophilus*	*E. coli* BL21 (DE3)	One-step; Ni-NTA affinity chromatography utilizing C-terminal His_6_ tag	0.151 (hydrolytic)	No transpeptidase activity	Castellano et al., 2011
7.	*Deinococcus radiodurans*	*E. coli* BL21 (DE3)	One-step; Ni-NTA affinity chromatography utilizing C-terminal His_6_ tag	0.060 (hydrolytic)	No transpeptidase activity	Castellano et al., 2011
8.	*E. coli*	*E. coli* BL21 (DE3)	One-step; Ni-NTA affinity chromatography utilizing N-terminal His_6_ tag	51.41 (U/ml)	High transpeptidase activity; high purification yield of 62 mg/L	Wang et al., 2011
9.	*Bacillus pumilus* KS12	*E. coli* BL21 (DE3)	One step; Ni-NTA affinity chromatography utilizing C-terminal His_6_ tag	1.82 (hydrolytic); 4.35 (transpeptidase)	High hydrolytic activity	Murty et al., 2012
10.	*Picrophilus torridus* DSM 9790	*E. coli* Rosetta	One-step; Ni-NTA affinity chromatography utilizing C-terminal His_6_ tag	18.92 (hydrolytic)	Addition of 2% hexadecane enhanced production ∼10 times; no transpeptidase activity	Rajput et al., 2013
11.	*Bacillus licheniformis* ER-15	*E. coli* BL21 (DE3)	Two-step; acetone precipitation and Q-sepharose anion exchange chromatography	4.58	10.2-fold purification with 28.8 % yield	Bindal and Gupta, 2014
12.	*Pseudomonas protegens* Pf-5	*E. coli* BL21 (DE3)	One-step; Ni-NTA affinity chromatography utilizing C-terminal His_6_ tag	31.06 (hydrolytic); 4.28 (transpeptidase)	High hydrolytic activity	Kushwaha and Srivastava, 2014
13.	*Pseudomonas fluorescence* PfT-1	*E. coli* BL21 (DE3)	One-step; Ni-NTA affinity chromatography utilizing C-terminal His_6_ tag	36.67 (hydrolytic); 5.45 (transpeptidase)	High hydrolytic activity	Kushwaha and Srivastava, 2014
14.	*Pseudomonas syringae*	*E. coli* Rosetta-gami B (DE3)	Two-step; DEAE cellulofine and Butyl toyopearl column chromatography	0.92 (transpeptidase)	Low purification yield (1.83%) due to protein instability	Prihanto et al., 2015a
15.	*Bacillus licheniformis*	*E. coli* BL21 (DE3)	One-step; Ni-NTA affinity chromatography utilizing N-terminal His_6_ tag	−	High protein yield of 150–200 mg/2 L	Kumari et al., 2017
16.	*Bacillus licheniformis* ER-15	*E. coli* BL21 (DE3)	−	9000 U/L (extracellular activity); 3000 U/L (intracellular activity)	75–80% extracellular translocation using native signal of the enzyme	Bindal et al., 2018
17.	*Bacillus subtilis 168*	Sporulation-deficient *B. subtilis* 168	Two-step; Gigapite and SOURCE 15Q PE column chromatography	7.49 (hydrolytic); 100 (transpeptidase)	15-fold higher expression; 30% purification yield	Minami et al., 2003b
18.	*Bacillus amyloliquefaciens* SMB469	*B. subtilis* KCTC 3135	−	24.7 (U/ml)	23 times higher expression	Lee et al., 2017
19.	*Bacillus amyloliquefaciens* BH072	*B. subtilis* 168	One-step; Ni-NTA affinity chromatography utilizing His_6_ tag	55	Use of dual signal peptide enhanced secretion to 1.6 times; high purification yield of 90 mg/L	Mu et al., 2019
20.	*Bacillus pumilus* ML413	*B. subtilis* 168	Two-step; acetone precipitation and Ni-NTA affinity chromatography utilizing His_6_ tag	18.65 (U/ml)	Addition of poly(T/A) tail to *ggt* mRNA and coexpression of PrsA lipoprotein resulted in 60% high GGT production and 2-fold enhanced extracellular expression	Yang T. et al., 2019

Bacterial GGTs are distantly related to plant and mammalian GGTs in terms of their biochemical characteristics (Castellano and Merlino, 2012), but large variations exist among bacterial homologues; for example, *Bacillus* GGTs are 20–35 times catalytically more efficient than EcGGT (Lyu et al., 2009). Extremophilic bacterial GGTs exhibit only hydrolytic activity suggesting them to be the ancient progenitors that evolved earliest with only hydrolytic activity, and later, transpeptidase activity was imparted to other mesophilic bacterial and eukaryotic GGTs (Castellano et al., 2010, 2011).

Biochemical properties of prokaryotic GGTs are not very diverse, and they optimally function at alkaline pH ranging from 8.0 to 11.0 with different pH optima for hydrolysis and transpeptidation reactions (Balakrishna and Prabhune, 2014; Morelli et al., 2014). Most bacterial GGTs are stable over a wide pH range (6.0–11.0) with maximum stability at alkaline pH range (Shuai et al., 2011; Murty et al., 2012; Bindal and Gupta, 2014; Mu et al., 2019); this is due to the alkaline pKa of GGT substrates, which is above pH 9.0. Moreover, the enzyme can be controlled to catalyze hydrolysis, transpeptidation, or autotranspeptidation simply by adjusting reaction pH (Morelli et al., 2014). Bacterial GGTs exhibit highly variable thermal stability (Castellano et al., 2010) and are reported to optimally catalyze reactions between 37 to 60°C (Castellano and Merlino, 2012). Inhibition in enzyme activity after addition of amino-acid-specific inhibitors like N-bromosuccinimide and phenylmethylsulfonyl fluoride suggested crucial involvement of a tryptophan and serine/threonine residue (Suzuki et al., 1986; Shuai et al., 2011; Bindal and Gupta, 2014). Cysteine-protease inhibitors like β-mercaptethanol, dithiothreitol, and iodoacetamide show only marginal inhibition due to lack of sulfhydryl groups (Moallic et al., 2006; Rajput et al., 2013). GGT-specific substrate analogs like DON and azaserine show complete inhibition (Bindal and Gupta, 2014; Kushwaha and Srivastava, 2014). Metal chelating agents ethylenediaminetetraacetic acid (EDTA) and ethylene glycol tetraacetic acid (EGTA) exhibit moderate inhibition, suggesting contribution of a metal ion in the active conformation of enzyme. The catalytic activity of some bacterial GGTs is reported to enhance with divalent cations such as Mg^2++^ and Ca^2+^; however, GGT is not considered to be a metallopeptidase and is sensitized by most of the heavy metal ions like Zn^2+^, Cd^2+^, Hg^2+^, and Pb^2+^ (Lin et al., 2006; Yao et al., 2006; Shuai et al., 2011; Kushwaha and Srivastava, 2014).

The determination of salt tolerance capacity of bacterial GGTs also characterizes them as halophilic and nonhalophilic. GtGGT is considered the most halotolerant, as it retains more than 90% of its hydrolytic activity in 4 M NaCl, followed by BsGGT retaining 86% of its hydrolytic activity at 3 M NaCl, while EcGGT lost 90% of its hydrolytic activity at the same salt concentration (Wada et al., 2010; Pica et al., 2013). BlGGT has also been reported to tolerate 4 M NaCl concentration without significant effect on its activity (Yang et al., 2011). The underlying mechanism for halotolerance has been reported to be associated with the presence of a relatively large number of negatively charged residues (aspartate and glutamate) on the solvent-exposed surfaces of GtGGT and BsGGT structures even at high salt concentrations resulting in increased water binding capacity, thus increasing solvation of the protein and preventing self-aggregation. EcGGT has also been reported to have acidic residues patches on its surface; however, they are not maintained at the surface under hypersaline conditions (Wada et al., 2010).

Bacterial GGTs exhibit broad substrate specificity for amino acids and other amine-group containing compounds like ethylamine, taurine, 3,4-dihydroxyphenylalanine (DOPA) as acceptors and can thus synthesize a variety of γ-glutamyl compounds utilized in food and pharmaceutical industries. Dipeptides like glycyl glycine and glycyl-L-alanine are fairly good acceptors for bacterial GGTs, with the former being the best (Ogawa et al., 1991; Moallic et al., 2006). Monomeric L-arginine and L-lysine seem to be good acceptors, while L-alanine, L-serine, L-glutamine, and L-glutamic acids have been reported to be poor acceptors (Lee et al., 2017); the propensity of hydrophobic and aromatic amino acids like L-methionine, L-phenylalanine, and L-DOPA is quite good compared to branched acceptors (Suzuki et al., 1986; Ogawa et al., 1991; Shuai et al., 2011). Interestingly, EcGGT poorly accepts D-amino acids, while *Bacillus* GGTs can accept both L- and D-isomers, thus showing relaxed stereoselectivity at the acceptor site (Ogawa et al., 1991; Hu et al., 2012).

Biophysical characterization of bacterial GGTs has been done to understand the molecular basis of enzyme stability. Temperature- and guanidine hydrochloride (GdnHCl)-induced unfolding of mature and precursor mimic (T399A mutant) forms of BlGGT using CD and emission fluorescence revealed that the mature form was structurally more stable and that both forms displayed an irreversible two-state pattern of thermal unfolding (Hung et al., 2011). A similar analysis showed that EcGGT was more sensitive toward thermal and chemical denaturation than BlGGT, which was more salt stable also (Yang et al., 2011). On the other hand, mature and precursor mutant (T353A) of GtGGT behaved differently; the thermal unfolding of both forms interestingly showed a three-state model including a stable intermediate species, and they exhibited remarkable temperature stability owing to strong electrostatic interactions but were sensitive toward GdnHCl (Pica et al., 2012). In another report, Ho et al. (2013) showed that heat-denatured EcGGT was renatured into active conformation upon incubation at 4°C, while BsGGT showed no such renaturation. They have suggested, based on biophysical characterization and native polyacrylamide gel electrophoresis (PAGE) analysis, that EcGGT dissociated into individual subunits upon heat treatment, which reassembled into active conformation at 4°C (Ho et al., 2013).

## Immobilization of Bacterial GGTs

Immobilization of industrially relevant enzymes has been suggested to offset the cost of the process utilizing such enzymes and in circumventing problems of enzyme destabilization (Mohamad et al., 2015). Bacterial GGTs like EcGGT, BsGGT, and BlGGT have been extensively explored for their potential as industrial biocatalysts in the synthesis of a wide variety of γ-glutamyl compounds like L-theanine, γ-glutamyl taurine, γ-D-glutamyl-l-tryptophan, etc., with huge biotechnological applicability (Suzuki et al., 2002a, 2004a; Wang et al., 2008; Bindal and Gupta, 2014). Thus, immobilization has been mainly focused on these three enzymes using various techniques ranging from simple entrapment method to covalent linkage with a suitable support matrix (Table 5) to reduce enzyme production cost and to increase enzyme reusability and stability. However, there are only a few reports of GGT enzyme immobilization that might be attributed to the heterodimeric composition of the enzyme that makes it difficult to remain in the active conformation after immobilization.

**TABLE 5:** Immobilization of bacterial gamma-glutamyl transpeptidases (GGTs) on different matrices.

S. No.	Enzyme	Immobilization matrix	Immobilization method	Cross-linking agent	Loading capacity/immobilization efficiency	Recyclability	Storage stability	Comments*	References
1.	EcGGT	Ca-alginate-k-carrageenan beads	Entrapment	−	1.5 mg enzyme/g of alginate	∼55% activity retained after 6 cycles	50% residual activity after 35 days of storage	Improve thermal stability at 40°C; improved storage stability	Hung et al., 2008
2.	BlGGT	Ca-alginate beads	Entrapment	−	40–45% activity recovered; 0.02 U/mg of beads	>90% activity retained after three cycles	Not mentioned	Improve thermal stability at 60°C with >80% residual activity after 15 min	Bindal and Gupta, 2014
3.	BlGGT	Amino silane coated iron magnetic nanoparticles	Covalent linkage	Glutaraldehyde	32.4 mg/g of support; 52.4% activity recovered	∼36% activity retained after 10 cycles	82% residual activity rafter 30 days of storage	Comparable thermal and storage stability	Chen et al., 2013
4.	BlGGT	HBPAA-modified magnetic nanoparticles	Covalent linkage	NHS/EDC	16.2 mg/g of support; 47.4% activity recovered	∼32% activity retained after 10 cycles	98% residual activity after 63 days of storage	Slightly improved pH stability in alkaline pH range; comparable thermal and storage stability	Juang et al., 2014
5.	BsGGT	APES modified and unmodified TiO_2_ whiskers	Adsorption	−	184 U/g of support (modified TiO_2_ whiskers)	72 and 80% for unmodified and modified TiO_2_ whiskers, respectively, after 21 cycles	Stable for 60 days at 4°C	Improved pH stability at alkaline pH range (modified);	Wang et al., 2014
6.	BlGGT	Chitosan microspheres	Covalent linkage	Glutaraldehyde	11.9 U/mg dry weight of support	90% activity retained after 10 cycles	85% residual activity after 32 days of storage	Active in broad pH range; improved thermal and salt stability	Bindal and Gupta, 2016
7.	BsGGT	Pharmalyte modified titania oxide whiskers	Adsorption	−	Not mentioned	∼85% after 21 cycles	Stable for 60 days at 4°C	Active in broad pH range; 23 times improved pH stability under alkaline conditions	Ni et al., 2017
8.	BlGGT	Graphene oxide nanosheets	Covalent and noncovalent linkage	Glutaraldehyde	34.7 mg/mg of support; 68.7% activity recovered	46% activity retained after 5 cycles (noncovalent); 45% activity retained after 9 cycles (covalent)	92% (noncovalent) and 97% (covalent) after 30 days of storage	Active in a broad pH range; improved thermal stability at 60°C	Lin et al., 2018

Heterologously expressed EcGGT and BlGGT have been immobilized successfully by entrapment methods using Ca-alginate-k-carrageenan and Ca-alginate, respectively, and have been used in theanine synthesis (Hung et al., 2008; Bindal and Gupta, 2014). Bacterial GGTs have also been immobilized on various nanoparticles like iron magnetic nanoparticles, titanium dioxide whiskers, and graphene oxide nanosheets to increase enzyme loading capacity as well as immobilization efficiency and recyclability. Chen et al. (2013) reported covalent immobilization of BlGGT on aldehyde-functionalized magnetic nanoparticles with an efficiency of around 52 and 36% activity retainment after 10 cycles (Chen et al., 2013). In another report, BlGGT was immobilized onto hyperbranched poly(amido acid) (HBPAA)-modified magnetic nanoparticles displaying high storage stability than free enzyme and recyclability up to 10 cycles with 32% activity retained (Juang et al., 2014). Recently, BlGGT has also been immobilized covalently on the highly biocompatible chitosan microsphere beads showing high recyclability with 90% activity retained even after 10 cycles along with improved thermal and salt stability (Bindal and Gupta, 2016).

Immobilization of BsGGT on unmodified and APES-modified mesoporous TiO2 whiskers (denoted as MTw and MTwA, respectively) improved its physicochemical properties. MTw-immobilized BsGGT displayed better thermal stability, while MTwA-immobilized BsGGT exhibited better pH stability in alkaline pH range beneficial for industrial use; as bacterial GGTs are reported to react optimally at alkaline pH (Wang et al., 2014). The modified MTwA BsGGT exhibited good operational stability with more than 80% activity retained after 21 cycles and storage of 60 days at 4°C as well as significantly higher loading capacity than unmodified MTw. In another study, BsGGT immobilized on mesoporous titania whiskers (MTWs) was further modified by adsorbing Pharmalyte on MWT (Ni et al., 2017). Pharmalyte with an isoelectric point of ∼9.2 formed a buffering layer around MTW-immobilized enzyme, thus protecting it from surrounding bulk liquid with significant improvement in pH stability of the immobilized enzyme under alkaline conditions. At an elevated pH 11.0, the pH stability of the immobilized enzyme was 23 times higher than the free enzyme.

Recently, Lin et al. (2018) reported a facile method of BlGGT immobilization on graphene oxide nanosheets by both covalent and noncovalent bonds. The noncovalently immobilized enzyme showed less operational stability with 46% activity remaining after five repeated cycles, while the covalently immobilized enzyme retained about 45% activity after nine cycles of usage with improved thermal stability (Lin et al., 2018). Further, the covalently immobilized enzyme was successfully used as a biocatalyst in synthesizing two γ-glutamyl peptides, *viz*γ-glutamyl-phenylalanine and γ-glutamyl-leucine.

## Biotechnological Applications of Bacterial GGTs

Bacterial GGTs generate an array of compounds through enzymatic γ-glutamylation of amino acids (e.g., Cys, Glu, Phe, Leu, Val, Trp, and His) and other related amine compounds [e.g., 3,4-dihydroxyphenylalanine (L-DOPA) and ethylamine] exhibiting value-added properties like increased solubility, protease resistance, enhanced flavors, and therapeutic capabilities making such compounds significant in food and pharmaceutical industries. Recently, Hideyuki Suzuki (2019) has briefly reviewed the application of bacterial GGTs in food and medicine sectors also (Suzuki, 2019).

### Increase in Solubility and Protease Resistance by γ-Glutamylation

Gamma-glutamylation of cysteine has been reported to increase its water-solubility by threefolds (Hara et al., 1992). In addition, γ-glutamylated glutamine exhibited high thermal stability as compared to glutamine, which gets readily converted to pyroglutamic acid in aqueous solutions, thus making the former suitable for nutritional and therapeutic use (Sayed et al., 2010).

Additionally, synthesis of prodrugs like γ-glutamyl 3,4-dihydroxyphenylalanine (L-DOPA) has been reported to have increased half-life due to resistance to cleavage by general serum proteases (Hara et al., 1992; Misicka et al., 1996). Gamma-glutamyl L-DOPA has been shown to increase organ-specific dopamine concentration in mouse models and is thus perceived a potential treatment for Parkinson’s disease (Ichinose et al., 1987). Moreover, γ-glutamyl L-DOPA has also been used as an assay substrate for clinical detection of GGT in human serum samples (Kiuchi et al., 1986). GGT from *P. mirabilis* and *E. coli* K-12 have been employed in enzymatic synthesis of γ-glutamyl L-DOPA with 6.7 and 79% conversion rates, respectively (Nakayama et al., 1985; Kumagai et al., 1988).

### Gamma-Glutamylation for Synthesis of Flavor-Enhancing Peptides

Some essential amino acids like Phe, Leu, Val, His, etc., taste bitter when consumed orally. The addition of γ-glutamyl moiety to these amino acids using *E. coli* K-12 GGT has been reported to dramatically reduce their bitterness and improve their taste preference due to the introduction of a refreshing sour flavor, making them suitable for oral consumption as amino acid supplements (Suzuki et al., 2002b, 2004b).

Recently, the importance of γ-glutamyl peptides in imparting kokumi taste to various food items has also been highlighted (Yang J. et al., 2019). Many kokumi flavor-enhancing γ-glutamyl dipeptides, tripeptides, and sulfur-containing compounds like γ-glutamyl-S-alk(en)yl-L-cysteine have been reported to occur naturally in edible legumes, garlic, onion, and several fermented foods like cheese, fish, yeast extract, and soy sauce (Kuroda et al., 2012; Liao et al., 2013; Liu et al., 2015). Enzymatic synthesis of such kokumi peptides has also been successful employing bacterial GGTs as biocatalysts with L-glutamine as a donor and various amino acids as acceptors (Table 6). Large-scale production of kokumi peptides using bacterial GGTs has been suggested to be a cost-effective and suitable alternative for their commercial use, thus not depending on their natural sources, which involve agricultural and environmental limitations (Yang J. et al., 2019).

**TABLE 6:** List of enzymatically synthesized flavor-enhancing peptides.

Enzyme source	Peptides	Application	References
*E. coli* GGT	γ-glutamyl phenylalanine; γ-glutamyl histidine; γ-glutamyl leucine; γ-glutamyl valine	Debittering of bitter amino acids	Suzuki et al., 2002b, 2004b
*B. subtilis* GGT	γ-glutamyl methionine;	Flavor-enhancing kokumi properties	Morelli et al., 2014
*B. licheniformis* GGT	γ-glutamyl phenylalanine; γ-glutamyl leucine; γ-L-glutamyl-S-allyl-L-cysteine	Flavor-enhancing kokumi properties	Chen et al., 2015; Chi et al., 2017; Lee et al., 2018
*B. amyloliquefaciens* GGT (L-glutaminase with transpeptidase activity)	γ-[Glu](1 ≦ *n* < 5)-phenylalanine γ-[Glu]n-valine; γ-[Glu]n-methionine γ-[Glu](*n* ≥ 1)-Gln	Short-chain peptides with kokumi flavor	Yang et al., 2017, 2018
*B. subtilis* GGT (salt tolerant)	γ-glutamyl valyl glycine; γ-glutamyl valine	Flavor-enhancing kokumi properties	Ho and Suzuki, 2013
*B. subtilis* GGT (salt tolerant)	Glutamic acid	Umami flavor to soy sauce	Kijima and Suzuki, 2007

The hydrolytic activity of bacterial GGTs has also been exploited in soy sauce fermentation for imparting a characteristic umami taste (Kijima and Suzuki, 2007). Traditionally, soy sauce is prepared by proteolytic digestion of soy protein by fungal proteases like those from *Aspergillus oryzae*/*sojae* (Ito et al., 2013). The umami flavor of soy sauce has been introduced by the conversion of free glutamine released during fermentation to glutamic acid by fungal glutaminases. However, inhibition of fungal glutaminases at high salt concentrations during fermentation leads to unwanted conversion of glutamine to pyroglutamic acid. Thus, a salt-tolerant glutaminase activity is crucial for soy sauce fermentation. For this, GGT from *B. subtilis* (Kijima and Suzuki, 2007) and *G. thermodenitrificans* (Castellano et al., 2010) have been discovered to be salt tolerant with more than 80 and 90% hydrolytic activity retained in the presence of 18% NaCl, respectively. As GGT from *Bacillus subtilis* shows both hydrolytic and transpeptidase activity, salt-tolerant GGT from extremophilic *G. thermodenitrificans*, harboring only hydrolytic activity, is a better alternative to glutaminases in soy sauce fermentation. However, salt-tolerant GGT from *B. subtilis* can be exploited in another Japanese traditional seasoning, meso, where both transpeptidase and hydrolytic activities are required in improving product taste (Ho and Suzuki, 2013).

Recently, Kalum et al. (United States 2020/0196617 A1) have filed a patent against the utilization of GGT enzyme from *B. licheniformis*/*Bacillus horikoshii* in dough relaxation (Kalum et al., 2020). They have claimed that the addition of bacterial GGT enzyme during dough making can result in better extensibility of dough, which would help in producing flattened dough during the manufacturing of products like bread, flatbread, crackers, pizzas, pasta, noodles, laminated baking products, biscuits, baguettes, and hamburgers.

### Nutraceutical and Therapeutic γ-Glutamyl Compounds

In nature, γ-glutamyl peptides are ubiquitously present in bacteria, plants, and animals. They are the products and by-products of the glutathione cycle. Many naturally occurring γ-glutamyl peptides and their derivatives like theanine, γ-D-glutamyl aminomethylphosphonate, γ-D-glutamylglycine, γ-D-glutamyltaurine, γ-D-glutamyl-β-alanine, etc., have been reported to be beneficial for human consumption (Suzuki et al., 2007; Yang J. et al., 2019). Among them, peptides like L-theanine, γ-glutamyl taurine, and γ-D-glutamyl-L-tryptophan have been extensively studied and synthesized using bacterial GGTs. However, in the future, in-depth study and analysis of other naturally occurring γ-glutamyl peptides and their prospective use in pharmaceuticals would also demand their enzymatic synthesis, which eventually would increase the biotechnological demand of bacterial GGTs.

### L-Theanine

L-Theanine (γ-L-glutamylethylamide) is a nonproteinogenic amino acid occurring naturally in tea. Besides imparting the characteristic umami flavor to tea, L-theanine has various neurophysiological benefits on human health like regulating high blood pressure (Yokogoshi et al., 1995), improving cognition power (Haskell et al., 2008), reducing stress and promoting relaxation (Lu et al., 2004; Kimura et al., 2007), strengthening the immune system (Miyagawa et al., 2008), and enhancing antitumor activity (Sadzuka et al., 2000; Liu et al., 2009) and antiobesity effects (Zheng et al., 2004).

Owing to such a wide array of health benefits, L-theanine has huge demand worldwide. However, due to shortcomings of natural extraction and chemical synthesis methods, scientists and industries have been moving toward the enzymatic synthesis of L-theanine (Mu et al., 2015). Bacterial enzymes like GGT, glutamine synthetase, L-glutaminase, and γ-glutamylmethylamide synthetase have been utilized in L-theanine synthesis. As compared to other enzymes, GGT has advantages like readily available enzyme source, convenient reaction set-up, short reaction time, and no ATP requirement (Mu et al., 2015).

There are numerous reports on the enzymatic synthesis of L-theanine using bacterial GGTs with variable conversion rates ranging from 27% to as high as 94% (Table 7). The percent conversion and yield of L-theanine obtained in different studies were observed to be highly influenced by the ratio of the donor (mainly L-glutamine) and acceptor (ethylamine) used in the reaction. In a recent report, N450A mutant of GGT from *B. licheniformis* showed a conversion rate of 94% as compared to 62% with wild type at pH 10.5 and 37°C after 4 h using substrate molar ratio of 1:2.4 corresponding to 250 mM L-glutamine and 600 mM ethylamine (Chi et al., 2019a). While in another report, a double mutant, V319A/S437G, of GGT from *B. amyloliquefaciens* resulted in an increased conversion rate for L-theanine synthesis from 58 to 83% at pH 10.5 and 37°C after 5 h using a substrate molar ratio of 1:10 (Li et al., 2020). Yang T. et al. (2019) demonstrated a fed-batch strategy to increase the yield of L-theanine employing GGT from *B. pumilus* overexpressed in *B. subtilis* 168 host. Addition of fixed amounts of glutamine and ethylamine after constant intervals resulted in a final yield of 53.5 g/L with a conversion rate of 63% after 16 h (Yang T. et al., 2019). Recently, the use of ultrasound power of 100 W has been reported to increase theanine yield by 2.61-fold using *E. coli* K-12 GGT with a conversion rate of 89.1% (Xu et al., 2020).

**TABLE 7:** Enzymatic synthesis of L-theanine using various bacterial gamma-glutamyl transpeptidases (GGTs) as biocatalysts.

Biocatalyst	L-Glutamine concentration (donor; mM)*	Ethylamine concentration (acceptor; mM)	Enzyme concentration (U/ml)	Reaction conditions	Percent conversion/yield^#^	References
*Pseudomonas nitroreducens* IFO 12694 L-glutaminase	700	1500	0.5	pH 11.0; 30°C; 7 h	39%; 38 g/L	Tachiki et al., 1998
*E. coli* K-12 GGT	200	1500	0.4	pH 10.0; 37°C; 2 h	60%; 120 mM	Suzuki et al., 2002a
*E. coli* Novablue GGT	10	40	1.04	pH 10.0; 37°C	40%	Yao et al., 2006
Ca-alginate-k-carrageenan immobilized *E. coli* Novablue GGT	25	40	1.5 mg/g alginate	pH 10.0; 40°C; 12 h	27%	Hung et al., 2008
Sodium alginate immobilized *E. coli* K-12 cells expressing GGT	300 (Glutamic acid γ-methyl ester)	3000	0.1 g/ml of immobilized cells	pH 10.0; 45°C; 18 h	69.3%	Zhang et al., 2010
*B. subtills* SK 11.004 GGT	20	50	0.06	pH 9.0; 37°C; 4 h	94%; 18.9 mM	Shuai et al., 2010
Sumo-tag fused *E. coli* GGT	267	2000	1.5	pH 10.5; 45°C; 24 h	80%; 41 g/L	Wang et al., 2011
*B. subtilis* NX-2 GGT	48 [L-glutamine-Zn(II) complex]	1600	0.5	pH 9.0; 37°C; 3 h	63.8%; 61.3 mM	Wang et al., 2012
*E. coli* GGT	10 (γ-glutamyl-p-nitroanilide)	200	−	pH 9.0; 37°C; 6 h	93%	Zhang et al., 2013
*P. nitroreducens* DSM 14399 L-glutaminase	300	1500	1.5 U/5 ml	pH 10.0; 37°C; 5 h	40%; 28 g/L	Pu et al., 2013
*B. licheniformis* ER 15 GGT	80	600	1	pH 9.0; 37°C; 4 h	>84%	Bindal and Gupta, 2014
GGT from *B. subtilis* 168	200	2200	−	pH 10.0; 37°C; 5 h	78%	Chen et al., 2014
*B. amyloliquefaciens* GGT	20	100	0.5	pH 9.0; 40°C; 6 h	48%	Mu et al., 2019
*B. pumilus* ML413 GGT	250	2500	1	pH 10.0; 30°C; 9 h (batch reaction)	74%; 32 g/L	Yang T. et al., 2019
*B. pumilus* ML413 GGT	250 (25 g/L added after every 3 h)	2500 (73 g/L added after every 3 h)	1	pH 10.0; 30°C; 16 h (fed batch reaction)	63%; 53.5 g/L	Yang T. et al., 2019
*B. licheniformis* N450A mutant GGT	250	600	25 μg/ml	pH 10.5; 37°C; 4 h	94%	Chi et al., 2019a
*B. licheniformis* N450D mutant GGT	250	600	25 μg/ml	pH 10.5; 37°C; 4 h	75%	Chi et al., 2019a
*B. licheniformis* GGT	250	600	25 μg/ml	pH 10.5; 37°C; 4 h	62%	Chi et al., 2019a
*P. nitroreducens SP.001* L-glutaminase	750	1000	1	pH 9.5; 30°C; 11 h	66.1%; 490 mM	Shuai et al., 2019
*Bacillus amyloliquefaciens* GGT mutant V319A/S437G	200	2000	20 μg/ml	pH 10.0; 37°C; 5 h	83%	Li et al., 2020
*E. coli* K-12 GGT	120	480		pH 9.0; 45°C; 5 h; 100 W ultrasonic waves	89.1%; 18.5 g/L	Xu et al., 2020

*Donor substrate used other than glutamine mentioned in bracket.#Percent conversion calculated against initial concentration of L-glutamine donor.

Interestingly, in a recent report by Sun et al. (2019), endophytic bacteria present in *Camellia sinensis* has been shown to contribute toward the production of L-theanine. The endophyte has been identified as *Luteibacter* spp., and its GGT enzyme has been demonstrated to exhibit high activity for L-theanine synthesis using L-glutamine and ethylamine as substrates. Thus, high conversion rate and high yields obtained using bacterial GGTs suggest their potential in bio-catalytic synthesis of L-theanine at an industrial scale for commercial use.

### Gamma-D-Glutamyl-L-Tryptophan

Gamma-D-glutamyl-L-tryptophan (also known as SCV-07 or Golotimod) is an immunomodulatory dipeptide with broad-spectrum stimulatory activities that boost immune response (Kolobov and Simbirtsev, 1999). It has been implicated as a potential medicine for the treatment of tuberculosis (Simbirtsev et al., 2003) and a potent antiviral therapeutic agent against genital HSV-2 recurrent disease (Rose et al., 2008). The role of this peptide in suppressing tumor growth and reducing severity and duration of oral mucositis induced by chemoradiation therapy has also been reported (Watkins et al., 2010; Alterovitz et al., 2011; Papkoff et al., 2011; Tuthill et al., 2011).

Enzymatic synthesis of SCV-07 can be a promising alternative to meet the future demand of this prospective drug. Bacterial GGTs can take both L- and D-forms of glutamine as donor and show high specificity toward aromatic amino acid L-tryptophan as acceptor and thus can be used as a biocatalyst. Bacterial GGTs from *E. coli* and *Bacillus* species have been employed successfully in enzymatic synthesis of SCV-07 (Suzuki et al., 2004a; Wang et al., 2008; Saini et al., 2017). GGT from *E. coli* could synthesize 33 mM product with a conversion rate of 66% using a 1:1 molar ratio of D-glutamine (donor; 50 mM) and L-tryptophan (acceptor; 50 mM) in 5 h at 37°C (Suzuki et al., 2004a). At similar reaction conditions, *B. atrophaeus* GGT led to 50% conversion within 6 h with a product yield of 25 mM (Saini et al., 2017). GGT from *B. subtilis* NX-2 catalyzed SCV-07 synthesis using a 5:7 molar ratio of D-glutamine and L-tryptophan with a conversion rate of 42% after 4 h at 40°C (Wang et al., 2008). However, *B. subtilis* NX-2 GGT also catalyzed irreversible hydrolysis of the product γ-D-glutamyl-L-tryptophan, which resulted in a lower conversion rate.

### Gamma-Glutamyl-Taurine

Gamma-glutamyl taurine has been isolated from brains of different mammals and suggested to be synthesized by mammalian GGT *in vivo* (Török et al., 1981; Marnela et al., 1985). Many physiological effects of γ-glutamyl taurine on the central nervous system have been reported (Davies et al., 1982; Bittner et al., 2005). It has also been reported to show a positive ionotropic effect on the locust heart (Feuer and S.-Rózsa, 1981), on metamorphosis of amphibians (Feuer et al., 1978), and on the activity and concentration of plasma renin (Feuer and Gaál, 1979). This naturally occurring peptide with numerous physiological benefits has been synthesized enzymatically under *in vitro* conditions. As mammalian GGTs are difficult to purify owing to their membrane-bound location and heavy glycosylation, bacterial GGTs are being employed for enzymatic synthesis of γ-glutamyl taurine (Suzuki et al., 2002c). GGT from *E. coli* catalyzed the synthesis of the peptide using 200 mM L-glutamine and 200 mM taurine and a conversion rate of 22.5% corresponding to 45 mM γ-L-glutamyl taurine was achieved after 1 h of reaction at 37°C. Lower conversion rates were attributed to a poor affinity for taurine as an acceptor and the formation of by-products like γ-glutamyl glutamine and γ-glutamyl-γ-glutamyl-taurine (Suzuki et al., 2002c). Interestingly, γ-D-glutamyl taurine, the isomeric form of γ-L-glutamyl taurine, has also been reported to have similar physiological effects to some extent (Davies et al., 1982; Jones et al., 1984). *E. coli* GGT has been reported to exhibit poor specificity toward D-amino acids as γ-glutamyl acceptors (Suzuki et al., 1986). Thus, it has been employed to synthesize γ-D-glutamyl taurine with a conversion rate of 71% using D-glutamine as a donor (Suzuki et al., 2003).

### Allied Applications of Microbial GGTs

In addition to transpeptidase and hydrolytic activities, a few microbial GGTs have also been reported to exhibit some unique activities like cephalosporin acylase (CA) and β-aspartyl transferase (BAT), as they can act on substrates like glutaryl-7-aminocephalosporanic acid and asparagine, respectively.

Cephalosporin acylases that catalyze the conversion of cephalosporin C to 7-aminocephalosporanic acid, a starting material for the synthesis of semisynthetic cephalosporins, exhibits a higher affinity for GGT substrate than CA substrate (Yamada et al., 2008). Thus, it has been speculated that class IV CAs are primarily GGTs with adventitious CA activity (Li et al., 1999). Microbial GGTs have also been explored for CA activity, and GGT from *B. subtilis* has been reported to possess an inherent CA activity, as it can catalyze deacylation of glutaryl-7-aminocephalosporanic acid (GL-7-ACA) and produce 7-aminocephalosporanic acid (Yamada et al., 2008). However, the activity reported was very low, which could be enhanced to 963-fold for a triple mutant E423Y/E442Q/D445N of BsGGT, developed by site-directed and random mutagenesis approach (Suzuki et al., 2010). In another study, a single-point mutation at residue 433 (D433N) introduced CA activity in *E. coli* GGT (EcGGT), which was initially absent (Suzuki et al., 2004c). Subsequent introduction of two random mutations, Y444A and G484A, to D433N EcGGT variant further enhanced catalytic efficiency for GL-7-ACA substrate by 50-fold (Yamada et al., 2008). Thus, microbial GGTs have the potential to be utilized in the pharmaceutical industry as CAs.

Gamma-glutamyl transpeptidase from *Pseudomonas syringae* (PsGGT) has been reported to utilize asparagine in addition to glutamine as a donor substrate and catalyze the transfer of β-aspartyl and γ-glutamyl moiety to hydroxylamine, respectively (Prihanto et al., 2015a). The transfer of β-aspartyl moiety was higher than the transfer of γ-glutamyl moiety, thus making it a novel GGT exhibiting both γ-glutamyl transferase and β-aspartyl transferase activities. Apart from PsGGT, only one enzyme from *Mycobacterium tuberculosis*, *viz* aspartotransferase, has been reported to exhibit β-aspartyl transferase activity (Reddy et al., 1969). Enzymes like asparaginase can only catalyze the hydrolysis of asparagine, but no transfer reaction has been reported (Hendriksen et al., 1969). The novel β-aspartyl transferase activity of PsGGT was applied for the synthesis of L-β-aspartyl hydroxamate (BAH) using 80 mM L-asparagine (donor substrate) and 40 mM hydroxylammonium chloride (acceptor substrate) with 0.106 mM product yield (Prihanto et al., 2015b).

In a recent report, degradation fragments of GGT from *B. subtilis* BU108 have been demonstrated to have antimicrobial activity against *Streptomyces scabiei* (Liu et al., 2020). Two antimicrobial peptides (AMPs), *viz* KT20 (KKGGNAIDAAVAIQFALNVT) and IF20 (IQKDLAKTFKLIRSNGTDAF), have been identified, which are the degradation fragments of GGT enzymes. Thus, *B. subtilis* GGT has been speculated to have an application in a potato common scab biocontrol.

## Conclusion and Future Perspectives

Gamma-glutamyl transpeptidase enzyme has been studied extensively over the past few decades owing to its universal existence and high sequence similarity among the prokaryotes and eukaryotes, indicating evolutionary conservation, the reason for which is not clear yet. Just like mammals, GGTs from Gram-negative bacteria also degrade glutathione; Gram-positive ones, however, are recognized as PGA degraders and hence grouped separately. GGT has many physiological roles in Gram-positive and Gram-negative bacteria (Table 1). Recently, the involvement of *H. pylori* GGT (HpGGT) in pathogenesis has been well established, and HpGGT can be explored as a potential drug target in combating *H. pylori* infection in combination with antibiotics. Likewise, extensive research on GGTs from other bacterial pathogens is required in understanding their virulence mechanism for developing efficient drugs.

Besides their physiological and clinical relevance, crystallization of bacterial GGTs also unveiled molecular aspects of autoprocessing and catalysis, emphasizing the role of conserved threonine that acts as a nucleophile. However, the interplay between small and large subunit formed after autoprocessing is yet to be elucidated. There are few reports on *in vitro* association of large and small subunit with variable results, as the decoupling of autoprocessing was only successful in a few cases. Crystallization studies have provided details on donor binding site, while identification of residues of acceptor binding site is still nebulous. Thus, crystallization should be attempted in the presence of acceptor substrate to have information about acceptor site residues. Mutational studies have been done focusing mainly on catalytically important small subunit residues to develop variants with improved catalytic activity. However, crystallization of such mutants is also required for a better understanding of structure and function.

Simpler handling of bacterial cultures, their easy genetic manipulations for overexpression, simpler purification techniques, and utilization of glutamine as a cheaper donor source make them a potential candidate for industrial exploitation. Most work has been done on the enzymatic synthesis of theanine, a nutraceutical with huge market potential, and numerous patents have been granted for the same (Table 8); however, still, no product enzymatically synthesized by GGT has been commercialized to date. Lack of commercially available GGTs could be one of the major reasons along with meager studies on the bioactivity of other potential γ-glutamyl peptides like γ-D-glutamyl-L-tryptophan, γ-glutamyl taurine, and other kokumi peptides. Addressing these issues along with immobilization techniques would aid in increasing the market value of GGTs. Other allied applications of bacterial GGTs showing unique β-aspartyl transferase activity and as a biocontrol agent require in-depth exploration to widen their applicability.

**TABLE 8:** List of patents on applications of bacterial gamma-glutamyl transpeptidases (GGTs).

S. No.	Patent No.	Title	Technology Organism/enzyme/substrates/conditions	Assignee/inventors	Filling date
1.	United States 20200196617 A1	Dough Relaxation Using Gamma Glutamyl Transpeptidase	Bacterial GGT is added to flour to make dough	Novozymes AS	2018-06-20
2.	US9512177B2	Method for producing γ-glutamyl-valyl-glycine crystal	Microbial GGT is used to convert valyl-glycine to γ-glutamyl-valyl-glycine	Ajinomoto Co Inc	2015-02-10
3.	*WO2006102722A1*	Process for the production of gamma-glutamylcysteine	Bovine GGT is used to synthesize gamma-glutamyl cysteine	Wallace John BridgeMartin Hani Zarka	2006-03-31
4.	JP8919992A	Production of l-gamma-glutamyl-lower alkylamide	Purified *Bacillus subtilis* GGT; L-glutamine and ethylamine hydrochloride; pH 10.0, 37°C and 18 h	Daiwa Kasei Kk	1992-04-10
5.	JP18431892A	Production of theanine	Immobilized *Pseudomonas nitroreducens* IFO 12694 cells; L-glutamine and ethylamine; pH 9.5 and 22 days	Taiyo Kagaku Co Ltd	1992-05-30
6.	CN101270376A	Method for synthesizing L-theanine with enzyme	Gamma-glutamyltranspeptidase; L-glutamine-copper (II) complex and ethylamine; pH 8.0–11.0, 35–45°C, 2–5 h	Car Taiyo Kagaku Kabushiki Kaisha	2008-05-14
7.	CN101343618A	Preparation method for natural theanine	GGT from *Bacillus subtilis* SK11.004 strain; glutamine and ethylamine; pH 9–10.5, 37°C and 2 h	Car Taiyo Kagaku Kabushiki Kaisha	2008-08-19
8.	CN101445787A	Method for biosynthesizing theanine by immobilized gene-engineering strain	Immobilized *E. coli* cells harboring pET32a-GGT plasmid; glutamine and ethylamine hydrochloride; pH 9–10, 32°C	Tea Research Institute of Chinese Academy of Agricultural Sciences	2008-12-04
9.	CN101445788A	Method for biosynthesizing theanine by gene-engineering strain	Recombinant *E. coli* GGT; glutamine and ethylamine hydrochloride; pH 9, 37°C	Tea Research Institute of Chinese Academy of Agricultural Sciences	2008-12-04
10.	US20090169704A1	Process for the enzymatic preparation of a gamma-glutamyl compound	GGT enzyme derived from a plant belonging to the Graminaceae or Leguminaceae family, or from *Camellia sinensis*; glutamine or glutamic acid; ethylamine or ethylamine hydrochloride; pH is < 8.0	ConopcoInc	2008-12-18
11.	CN101457241B	Method for preparing theanine by using species coupling ATP regenerative technology	GGT from immobilized *Bacillus licheniformis* cells and ATP regeneration from *Bacillus stearothermophilus*; glutamine (glutamic acid) and ethylamine; pH 10. 5, 30°C, 48 h	Tea Research Institute of Chinese Academy of Agricultural Sciences	2009-01-04
12.	CN101560532A	L-theanine enzymatic transformation preparation method	GGT enzyme or *Bacillus subtilis* whole cells; L-glutamic acid-gamma-alkyl ester or L-glutamyl hydrazide and ethylamine; pH 9–11, 25-60°C	Tea Research Institute of Chinese Academy of Agricultural Sciences	2009-05-25
13.	CN101643712B	Escherichia coli strain for efficiently converting glutamine to synthesize L-theanine and application thereof	*E. coli* of CCTCC No. M 209166; glutamine and ethylamine; 20–37°C, 40–48 h		2009-09-15
14.	CN103409475A	Method for synthesizing L-theanine through enzyme process	Recombinant GGT from *Bacillus subtilis* 168; glutamine and ethylamine; pH 9.5–10.5, 37–50°C, 2–5 h		2013-07-18
15.	CN104372046A	Method for producing L-theanine by supplementing material	GGT from *Bacillus subtilis*; glutamine and ethylamine hydrochloride; pH 9–11, 30–45°C, 10–20 h; substrates are supplemented after every 2 h		2014-11-19
16.	CN104404075A	Method for catalyzing to generate L-theanine by using recombinant *Bacillus subtilis* secreted gamma-glutamyltranspeptidase	Recombinant *Bacillus subtilis* GGT expressed in *Bacillus subtilis* expression host; glutamine and ethylamine hydrochloride; pH 10, 37°C, 5 h		2014-12-09
17.	CN104561160A	Method for preparing theanine by using biological method	*Pseudomonas nitroreducens* cells; glutamine and triethylamine hydrochloride; pH 5.5–6.8, 25–38°C, 4–6h; L-glutamine is supplemented 1–3 times.		2014-12-22
18.	CN104789538A	Supplementary strategy for improving catalytic synthesis of L-theanine from gamma-glutamyltranspeptidase	Recombinant *Bacillus subtilis* GGT expressed in *Bacillus subtilis* expression host; glutamine and ethylamine hydrochloride; pH 10, 37°C, 18 h; glutamine is supplemented after regular intervals.		2015-03-30

Based on the present review, addressing bacterial GGTs and their relevance, it will be appropriate to put forth that detailed understanding of the structure–function relationship of bacterial GGTs utilized in synthesizing γ-glutamyl compounds has led to their emergence as the industrial biocatalyst in the current century and can be utilized in many other biotechnological sectors. It indicates that GGT is indeed an emerging biocatalyst, and in the coming decade, its commercial exploitation and new insights of its catalytic mechanism regarding acceptor binding can be visualized.

## Author Contributions

MS prepared the complete first draft along with conceptualization and review outline prepared in supervision of the corresponding author RG. AK contributed in finalization of a major portion of the review covering structural and functional aspects of bacterial GGTs and also contributed in finalizing bibliography. SB and KS contributed in finalizing parts of the manuscript covering biotechnological applications and physiological significance of bacterial GGTs. RG contributed during different stages of manuscript preparation starting from drawing the review outline to checking drafts at every stage for technical corrections and improving overall language for finalizing the manuscript. All authors contributed to the article and approved the submitted version.

## Conflict of Interest

The authors declare that the research was conducted in the absence of any commercial or financial relationships that could be construed as a potential conflict of interest.
