# Supplementary material for: Bacterial Gamma-Glutamyl Transpeptidase, an Emerging Biocatalyst: Insights Into Structure–Function Relationship and Its Biotechnological Applications
Source: Front Microbiol. 2021 Apr 9;12:641251. doi: 10.3389/fmicb.2021.641251 (PMC8062742; doi:10.3389/fmicb.2021.641251)
Supplement: Supplementary file 1 [file Data_Sheet_1.PDF]

```
BlGGT                                                   β1     β2
                      1         10        20        30        40        50
BlGGT          ......MRRLAFLVVAFCLAVGCFFSPVSKAEGVMS.GGDGDKVAVGKDGMVATAHPLAS
BpGGT          MEGVLFMKRISLTVLSICLFVFSFFLPVSQVTA..N.ETHGNKVAVGKDGMVATAHPLAS
BsGGT          ..MKRTWNVCLTALLSVLLVAGSVPFHAEAKKPPKS.YDEYKQVDVGKDGMVATAHPLAS
BaGGT          ..MKKTWNLCLTVLLSVMLAAGIAPFQAEAKKPPKN.YDEYTQVDVGKDGMVATAHPLAS
BamyGGT        MKKKKFMNLCFIVLLSTLLAAGSIPYHAQAKKHPFS.YDDYKQVDVGKDGMVATAHPLAS
EcGGT          MIKPTFLRRVAIAAL.LSGSCFSAAAAPPAPPVSYGVEEDVFHPVRAKQGMVASVDATAT
HpGGT          .MRRSFLKTIGLGVIALSLGLLSPLSAASYPPIK...........NTKVGLALSSHPLAT
PnGGT          ......MRVFHFSKLPLGVAILAASSSVFA...............TLDGGAVAAPDQYGA
HsGGT          MKKKLVVLGLLAVVLVLVIVGLCLWLPSASKEPDNH.........VYTRAAVAADAKQCS
CapD           ....................MGGIGVSCSFNKIKDSVKQKIDSMGDKGTYGVSASHPLAV
GtGGT          ..............................MDYLYHPYPSQRMTVFAKNGIVATSQPLAA
BhGGT          ..........................MSVMFDPQSYPYPSRRNVVYAKNGMVATSQPLAA
DrGGT          ...............................MTHNPEYPVVRRPAYARRGMVATSQPLAA
TtGGT          ..............................MDLTYYPYPSRRHVVLGRRGAVATSQPLAA
TaGGT          .....................................MFRSRPNALSQRSVIASSSELAS
PtGGT          .............................................MYMNYAVASSHPLST
consensus>50   ...............................................kdgmvatshpla.

BlGGT             α1             α2                  TT    β3         β4
                     60        70        80        90        100       110
BlGGT          KIGAEVLKKGGNAIDAAIAIQYALNVTEPMMSGIGGGGFMMVYDGETKETSIINSRERAP
BpGGT          EIGADVLKKGGNAVDAAVAIQYALNVTEPMMSGIGGGGFMMVYDGKTKETSIINSRERAP
BsGGT          EIGADVLKKGGNAIDAAVAIQFALNVTEPMMSGIGGGGFMMVYDGKTKDTTIIDSRERAP
BaGGT          EIGAEVLKKGGNAIDASVAIQFALNVVEPMMSGIGGGGFMMVYDGKTKDTTIINSRERAP
BamyGGT        QIGADVLKKGGNAIDAAVAIQFALNVTEPMMSGIGGGGFMMVYDAKTKDTTIIDSRERAP
EcGGT          QVGVDILKEGGNAVDAAVAVGYALAVTHPQAGNLGGGGFMLIR.SKNGNTTAIDFREMAP
HpGGT          EIGQKVLEDGGNAIDAAVAMGFALAVVHPAAGNIGGGGFAVIH.LANGENVALDFREKAP
PnGGT          KVAAEILKKGGNAVDAAVATAFTLAVTYPEAGNIGGGGFMTLY.V.DGKPYFLDYREIAP
HsGGT          KIGRDALRDGGSAVDAAIAALLCVGLMNAHSMGIGGGLFLTIYNSTTRKAEVINAREVAP
CapD           EEGMKVLKNGGSAVDAAIVVSYVLGVVELHASGIGGGGGMLI..ISKDKETFIDYRETTP
GtGGT          QAGLEVLKKGGNAIDAAIATAACLTVVEPTSNGIGGDAFALVW.T.NGKLYGLNASGYAP
BhGGT          QAGLDILKAGGNAIDAAIATATVLTVLEPTSNGIGSDAFALVW.T.KGKLHGLNGSGRAP
DrGGT          QAGLSILQAGGNAVDAAVATAAALTVVEPTSNGLGGDAFALVW.A.GGELHGLNASGAAP
TtGGT          LAGMEVLLKGGSAVDAAIAMAACLTVVEPTSNGIGGDLFALVW...DGTLHGLNASGKSP
TaGGT          LAGRDILKRGGNIFDAALAVSAMLCVTQNNLCGLGGDLFALIRDE.NGQIMDLNGSGQAS
PtGGT          FVGNEILKDGGNAYDAAIATSAALVVVQPHLNGLGGDFFSTI..I.DNDIYSINGSGNAP
consensus>50   eig.dvLkkGGnaiDAavai.fal.v.ep...giGgggfmlv.....ge...i#.re.ap

BlGGT          TT   η1     TT        α3      η2         α4               α5
                     120       130       140       150       160       170
BlGGT          EGAKPDMFLDEDGKVIPFSERSRHGNAVGVPGTLKGLEAAHKKWGTKKMEDLISPSIKLA
BpGGT          QGATPDMFLTDDGKVIPFAERSTHGNAVGVPGTVKGLEAALDKWGTRSMKELIEPSIQLA
BsGGT          AGATPDMFLDENGKAIPFSERVTKGTAVGVPGTLKGLEEALDKWGTRSMKQLITPSIKLA
BaGGT          AGATPDMFLDENGKAIPFSERVTKGTAVGVPGTLKSLEKALDMWGTRSMRQLIAPSIKLA
BamyGGT        AGATPDMFLDENGKAIPFSERVTKGTAVGVPGTLKGLEKALDKWGTRSMKQLITPSIKLA
EcGGT          AKATRDMFLDDQGNPDS.KKSLTSHLASGTPGTVAGFSLALDKYGTMPLNKVVQPAFKLA
HpGGT          LKATKNMFLDKQGNVVP.KLSEDGYLAAGVPGTVAGMEAMLKKYGTKKLSQLIDPAIKLA
PnGGT          KAATKTMYLNEKGEVIE.NLSLVGAKAAGVPGTVMGLWEAHQRFGKLKWSELLTPAIGYA
HsGGT          RLAFATMFNSSE.......QSQKGGLSVAVPGEIRGYELAHQRHGRLPWARLFQPSIQLA
CapD           .....YFTGNQ.............KPHIGVPGFVAGMEYIHDNYGSLPMGELLQPAINYA
GtGGT          AAISLDVLKERGYT.EM...PKYGFAPVTVPGAPAAWAALSKRFGRLSLAETLAPAIAYA
BhGGT          MSLTMEAVKAKGYEQEL...PPYGVIPVTVPGAPGAWAELAKMYGNLPLAASLAPAIRYA
DrGGT          AALSLEALPGG....EM...PKYGWTPVTVPGAVRGWTDLHGRFGRLDFAQVLAPAIRYA
TtGGT          MALTPERLPGR.....M...PERGWLPVTVPGAVSGWRALHERWGRLPFAEVLAPAIRYA
TaGGT          RAVSIDYYESMGLTKIP...ERGPYAAITVPGIAGSWDEIFRKFATMDIADILEPAIRTA
PtGGT          ELATIEFFHRNGYNKIP...EQGPLSSFSIPGLVSSWEILYKN.ATMKLEKLFSRAISFA
consensus>50   ..at.dmfldeng..ip.........avgvPGtv.gle.a.d.ygtl.m.el..pai.lA

BlGGT             β5     α6       α7                    α8        TT β6   α9
                     180       190       200       210       220
BlGGT          EEGFPIDSVLADAIKDHQDKL.......SKTAAKDIFLPDGEPLKEGDILVQKDLAKTFK
BpGGT          EDGFEIDSVLAKAIDDHQGKL.......KKTAAAPIFLPNDQPLEEGDLLVQPGLAKTFK
BsGGT          EKGFPIDSVLAEAISDYQEKL.......SRTAAKDVFLPNGEPLKEGDTLIQKDLAKTFK
BaGGT          AKGFPIDSVLAEAISDYQDKL.......SRTAAKDVFLPNGEPLKEGDKLVQKDLAKTFK
BamyGGT        SKGFPIDSVLADAISDYKDKL.......SHTAAKDVFLPDGEPLKEGDTLIQKDLAKTFT
EcGGT          RDGFIVNDALADDLKTYGSEVL.....PNHENSKAIFWKEGEPLKKGDTLVQANLAKSLE
HpGGT          ENGYAISQRQAETLKEARERFL.....KYSSSKYFFKKGHLDYQEGDLFVQKDLAKTLN
PnGGT          QTGFKVADQQYQYRQDAIALF..........NGKTNFGDYFGTMKPGEVFKQPELAKTLE
HsGGT          RQGFPVGKGLAAALENKRTVI......EQQPVLCEVFCRDRKVLREGERLTLPQLADTYE
CapD           EKGFKVDDSLTMRLDLAKPRI........YSDKLSIFYPNGEPIETGETLIQTDLARTLK
GtGGT          ENGYPVSPVLGKYWANAYRVYKEALHGPEFGSWFETFAPAGRAPNIGEVWASPDHAATLR
BhGGT          EEGYPVTPTLAKYWKAAYDRFKTEWTDDVYQPWFDTFAPKGRAPRVGEVWRSQGHADTLR
DrGGT          REGYPLSPVLAANWARAIRSY.WALNLPIFEDWFRTFAPDGFTPRPGALWRSEGHARTLE
TtGGT          EEGFPVGPETARSWRRAEGVF.LPLEGPEFGPFKEVFFPGGRAPRAGEVWRSPLHAKTLR
TaGGT          SAGFPITQNYSDSIARSAPVI......GQYRGWSSIFMPNGSVPVAGEILKQPDLAESFR
PtGGT          MDGFIPSNSILKAIKNFKYGD.........VDFNNIYYNNER......LLVQRALGKTLK
consensus>50   eeG%pi...lad...d.................kdi%.png..l..gd.lvq.dlaktl.
```

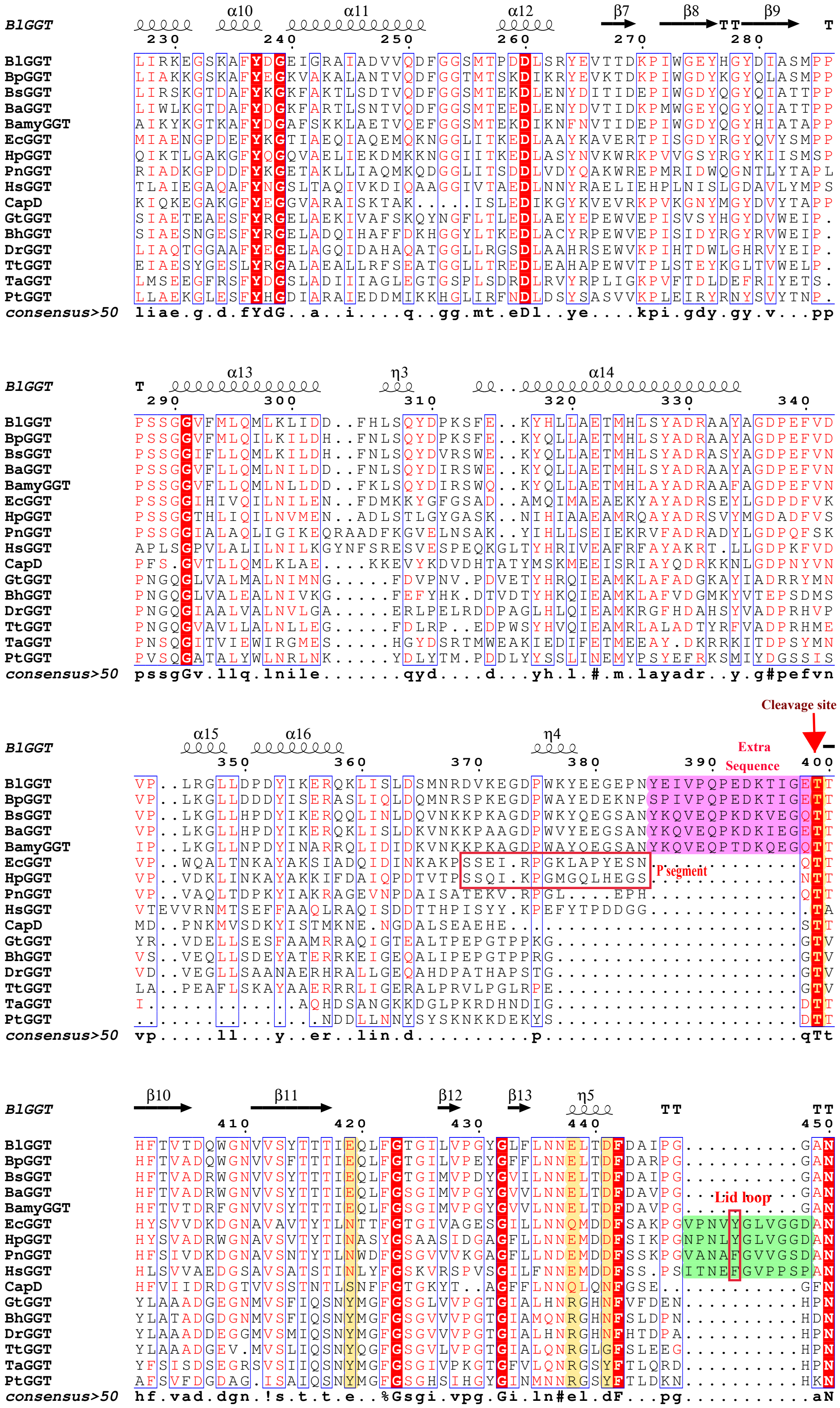
B1GGT α10 α11 α12 β7 β8 TT β9 T
230 240 250 260 270 280
B1GGT LIRKEGSKAFYDGEIGRAIADVVQDFGGSMTPDDLSRYEVTTDKPIWGEYHGYDIASMPP
BpGGT LIAKKGSKAFYEGKVAKALANTVQDFGGTMTSKDIKRYEVKTDKPIWGDYKGYQLASMPP
BsGGT LIRSKGTDAFYKGKFAKTLSDTVQDFGGSMTEKDLENYDITIDEPIWGDYQGYQIATTPP
BaGGT LIWLKGTDAFYDGKFARTLSNTVQDFGGSMTEEDLENYDVTIDKPMWGEYQGYQIATTPP
BamyGGT AIKYKGTKAFYDGAFSKKLAETVQEFGGSMTEKDIKNFNVTIDEPIWGDYQGYHIATAPP
EcGGT MIAENGPDEFYKGTIAEQIAQEMQKNGGLITKEDLAAYKAVERTPISGDYRGYQVYSMPP
HpGGT QIKTLGAKGFYQGQVAELIEKDMKKNGGIITKEDLASYNVKWRKPVVGSYRGYKIISMSP
PnGGT RIADKGPDDFYKGETAKLLIAQMKQDGGLITSDDLVDYQAKWREPMRIDWQGNTLYTAPL
HsGGT TLAIEGAQAFYNGSLTAQIVKDIQAAGGIVTAEDLNNYRAELIEHPLNISLGDAVLYMPS
CapD KIQKEGAKGFYEGGVARAISKTAK.....ISLEDIKGYKVEVRKPVKGNYMGYDVYTAPP
GtGGT SIAETEAESFYRGELAEKIVAFSKQYNGFLTLEDLAEYEPEWVEPISVSYHGYDVWEIP.
BhGGT SIAESNGESFYRGELADQIHAFFDKHGGYLTKEDLACYRPEWVEPISIDYRGYRVWEIP.
DrGGT LIAQTGGAAFYEGELAGQIDAHAQATGGLLRGSDLAAHRSEWVKPIHTDWLGHRVYEIP.
TtGGT EIAESYGESLYRGALAEALLRFSEATGGLLTREDLEAHAPEWVTPLSTEYKGLTVWELP.
TaGGT LMSEEGFRSFYDGSLADIIAGLEGTGSPLSDRDLRVYRPLIGKPVFTDLDEFRIYETS.
PtGGT LLAEKGLESFYHGDIARAIEDDMIKKHGLIRFNDLDSYSASVVKPLEIRYRNYSVYTNP.
consensus>50 liae.g.d.fYdG..a..i....q..gg.mt.eDl..ye....kpi.gdy.gy.v...pp
B1GGT T α13 η3 α14
290 300 310 320 330 340
B1GGT PSSGGVFMLQMLKLIDD..FHLSQYDPKSFE..KYHLLAETMHLSYADRAAYAGDPEFVD
BpGGT PSSGGVFMLQILKILDH..FNLSQYDPKSFE..KYQLLAETMHLSYADRAAYAGDPEFVD
BsGGT PSSGGIFLLQMLKILDH..FNLSQYDVRSWE..KYQLLAETMHLSYADRASYAGDPEFVN
BaGGT PSSGGVFLLQMLNILDD..FNLSQYDIRSWE..KYQLLAETMHLSYADRAAYAGDPEFVN
BamyGGT PSSGGVFLLQMLNLLDD..FKLSQYDIRSWQ..KYQLLAETMHLAYADRAAFAGDPEFVN
EcGGT PSSGGIHIVQILNILEN..FDMKKYGFGSAD..AMQIMAEAEKYAYADRSEYLGDPDFVK
HpGGT PSSGGTHLIQILNVMEN..ADLSTLGYGASK..NIHIAAEAMRQAYADRSVYMGDADFVS
PnGGT PSSGGIALAQLIGIKEQRAADFKGVELNSAK..YIHLLSEIEKRVFADRADYLGDPQFSK
HsGGT APLSGPVLALILNILKGYNFSRESVESPEQKGLTYHRIVEAFRFAYAKRT.LLGDPKFVD
CapD PFS.GVTLLQMLKLAE....KKEVYKDVDHTATYMSKMEEISRIAYQDRKKNLGDPNYVN
GtGGT PNGQGLVALMALNIMNG.....FDVPNV.PDVETYHRQIEAMKLAFADGKAYIADRRYMN
BhGGT PNGQGLVALEALNIVKG.....FEFYHK.DTVDTYHKQIEAMKLAFVDGMKYVTEPSDMS
DrGGT PNGQGIAALVALNVLGA.....ERLPELRDDPAGLHLQIEAMKRGFHDAHSYVADPRHVP
TtGGT PNGQGVAVLLALNLLEG.....FDLRP..EDPWSYHVQIEAMRLALADTYRFVADPRHME
TaGGT PNSQGITVIEWIRGMES.....HGYDSRTMWEAKIEDIFETMEEAY.DKRRKITDPSYMN
PtGGT PVSQGATALYWLNRLNK.....YDLYTM.PDDLYSSLINEMYPSYEFRKSMIYDGSSIS
consensus>50 pssgGv.llq.lnile.......qyd....d...yh.l.#.m.layadr..y.g#pefvn
Cleavage site
B1GGT α15 α16 η4 Extra Sequence
350 360 370 380 390 400
B1GGT VP..LRGLLDPDYIKERQKLISLDSMNRDVKEGDPWKYEEGEPNYEIVPQPEDKTIGETT
BpGGT VP..LKGLLDDDYISERASLIQLDQMNRSPKEGDPWAYEDEKNPSPIVPQPEDKTIGETT
BsGGT VP..LKGLLHPDYIKERQQLINLDQVNKKPKAGDPWKYQEGSANYKQVEQPKDKVEGQTT
BaGGT VP..LKGLLHPDYIKERQKLISLDKVNKKPAAGDPWVYEEGSANYKQVEQPKDKIEGETT
BamyGGT IP..LKGLLNPDYINARRQLIDIDKVNKKPKAGDPWAYQEGSANYKQVEQPTDKQEGQTT
EcGGT VP..WQALTNKAYAKSIADQIDINKAKPSSEI.RPGKLAPYESN.............QTT
HpGGT VP..VDKLINKAYAKKIFDAIQPDTVTPSSQI.KPGMGQLHEGS.............NTT
P'segment
PnGGT VP..VAQLTDPKYIAKRAGEVNPDAISATEKV.RPGL....EPH.............QTT
HsGGT VTEVVRNMTSEFFAAQLRAQISDDTTHPISYY.KPEFYTPDDGG..............TA
CapD MD..PNKMVSDKYISTMKNE.NGDALSEAEHE.........................STT
GtGGT YR..VDELLSESFAAMRRAQIGTEALTPEPGTPPKG.....................GTV
BhGGT VS..VEQLLSDEYATERRKEIGEQALIPEPGTPPRG.....................GTV
DrGGT VD..VEGLLSAANAERHRALLGEQAHDPATHAPSTG.....................GTV
TtGGT LA..PEAFLSKAYAAERRRLIGERALPRVLPGLRPE.....................GTV
TaGGT I.........AQHDSANGKKDGLPKRDHNDIG.....................DTT
PtGGT ..........NDDLLNNYSYSKNKKDEKYS.....................DTT
consensus>50 vp.....ll...y..er..lin.d..........p.....................qTt
B1GGT β10 β11 β12 β13 η5 TT TT
410 420 430 440 450
B1GGT HFTVTDQWGNVVSYTTTIEQLFGTGILVPGYGLFLNNELTDFDAIPG..........GAN
BpGGT HFTVADQWGNVVSFTTTIEQLFGTGILVPEYGFFLNNELTDFDARPG..........GAN
BsGGT HFTVADRWGNVVSYTTTIEQLFGSGIMVPDYGVILNNELTDFDAIPG..........GAN
BaGGT HFTVADRWGNVVSYTTTIEQLFGSGIMVPGYGVVLNNELTDFDAVPG..........GAN
Lid loop
BamyGGT HFTVTDRFGNVVSYTTTIEQLFGSGIMVPGYGVVLNNELTDFDAVPG..........GAN
EcGGT HYSVVDKDGNAVAVTYTLNTTFGTGIVAGESGILLNNQMDDFSAKPGVPNVYGLVGGDAN
HpGGT HYSVADRWGNAVSVTYTINASYGSAASIDGAGFLLNNEMDDFSIKPGNPNLYGLVGGDAN
PnGGT HFSIVDKDGNAVSNTYTLNWDFGSGVVVKGAGFLLNDEMDDFSSKPGVANAFGVVGSDAN
HsGGT HLSVVAEDGSAVSATSTINLYFGSKVRSPVSGILFNNEMDDFSS.PSITNEFGVPPSPAN
CapD HFVIIDRDGTVVSSTNTLSNFFGTGKYT..AGFFLNNQLQNFGSE...........GFN
GtGGT YLAAADGEGNMVSFIQSNYMGFGSGLVVPGTGIALHNRGHNFVFDEN..........HPN
BhGGT YLATADGDGNMVSFIQSNYMGFGSGVVVPGTGIAMQNRGHNFSLDPN..........HDN
DrGGT YLAAADEGGMVSMIQSNYMGFGSGVVVPGTGIALHNRGHNFHTDPA..........HPN
TtGGT YLAAADGEV.MVSLIQSNYQGFGSGVLVPGTGIALQNRGLGFSLEEG..........HPN
TaGGT YFSISDSEGRSVSIIQSNYMGFGSGIVPKGTGFVLQNRGSYFTLQRD..........HPN
PtGGT AFSVFDGDAG.ISAIQSNYMGFGSGHSIHGYGINMNNRGSYFTLDKN..........HKN
consensus>50 hf.vad.dgn.!s.t.t.e..%Gsgi.vpg.Gi.ln#el.dF...pg..........aN

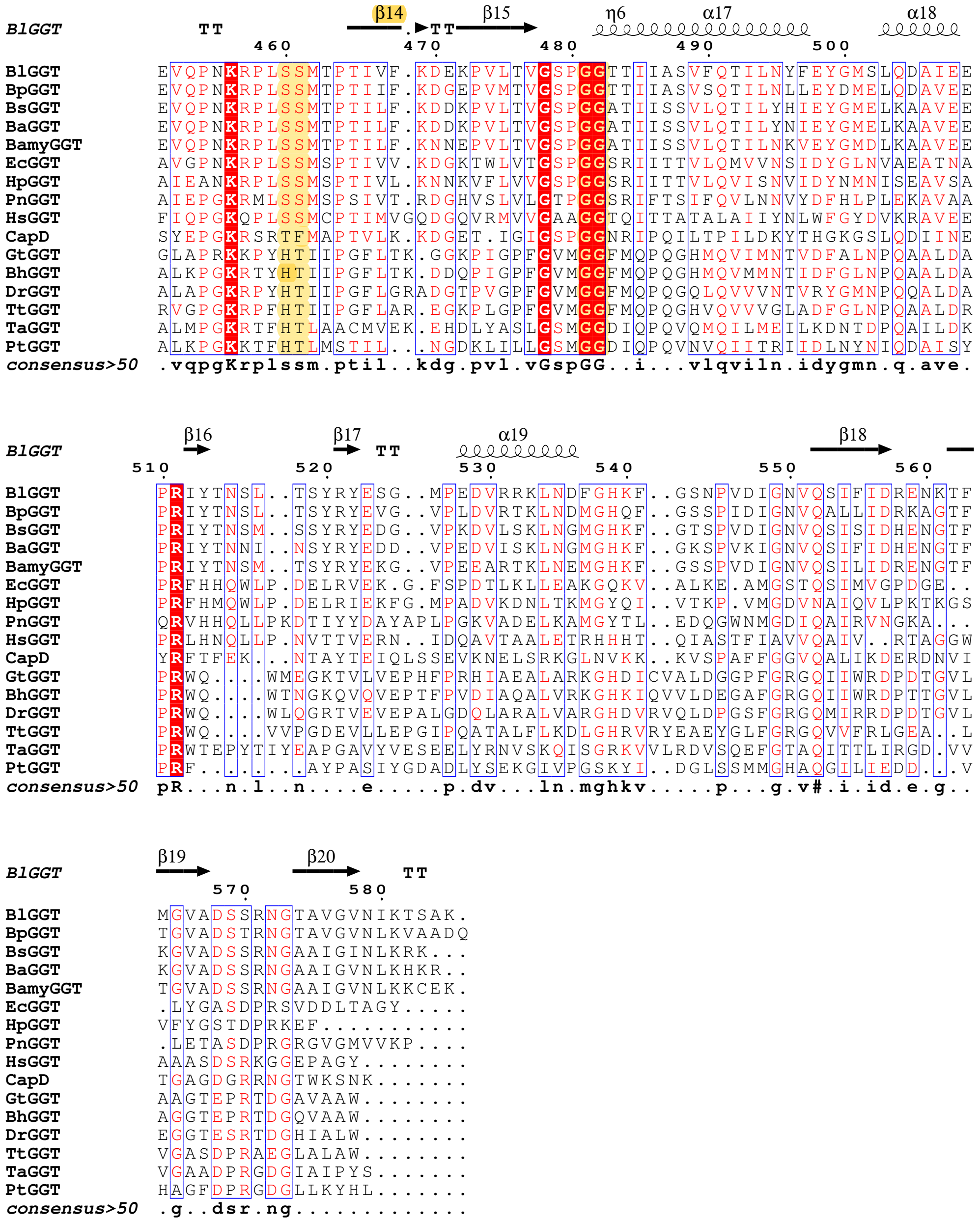

Supplementary Figure 1. Primary sequence alignment of different prokaryotic GGTs alongwith human GGT from mammals. BlGGT for *B. licheniformis* GGT (AAU22915.2); BpGGT for *B. pumilus* GGT (AHL71064.1); BsGGT for *B. subtilis* GGT (CAB13724.1); BaGGT for *B. atrophaeus* GGT (AKL84709.1); BamyGGT for *B. amyloliquefaciens* GGT (AGZ56610.1); EcGGT for *E. coli* GGT (CQR82862.1); HpGGT for *H. pylori* GGT (WP_001922043.1); PnGGT for *Pseudomonas nitroreducens* GGT (BAJ16340.1); HsGGT for *Homo sapiens* GGT (AAA52546.1); CapD for *Bacillus anthracis* GGT (WP_000508800.1); GtGGT for *Geobacillus thermodenitrificans* GGT (ARA98303.1); BhGGT for *B. halodurans* GGT (TES51851.1); DrGGT for *Deinococcus radiodurans* GGT (ANC71349.1); TtGGT for *Thermus thermophilus* GGT (AAS80751.1); TaGGT for *Thermoplasma acidophilum* GGT (WP_010901405.1); PtGGT for *Picrophilus torridus* GGT (AAT43770.1). Signal peptides present in some GGTs have been underlined in red; catalytic residues highlighted in yellow; cleavage site for autoprocessing indicated by arrow; lid loop region highlighted in green with an important aromatic residue shown in box; extra sequence region highlighted in purple and P-segment also indicated
